# Long-term trends in incidence and risk factors for ischaemic stroke subtypes: Prospective population study of the South London Stroke Register

**DOI:** 10.1371/journal.pmed.1002669

**Published:** 2018-10-05

**Authors:** Hatem A. Wafa, Charles D. A. Wolfe, Anthony Rudd, Yanzhong Wang

**Affiliations:** 1 School of Population Health and Environmental Sciences, King’s College London, London, United Kingdom; 2 National Institute for Health Research (NIHR) Biomedical Research Centre, Guy’s and St Thomas’ NHS Foundation Trust and King’s College London, London, United Kingdom; 3 National Institute for Health Research (NIHR) Collaboration for Leadership in Applied Health Research and Care (CLAHRC) South London, London, United Kingdom; Columbia University, UNITED STATES

## Abstract

**Background:**

As the average life expectancy increases, more people are predicted to have strokes. Recent studies have shown an increasing incidence in certain types of cerebral infarction. We aimed to estimate time trends in incidence, prior risk factors, and use of preventive treatments for ischaemic stroke (IS) aetiological subtypes and to ascertain any demographic disparities.

**Methods and findings:**

Population-based data from the South London Stroke Register (SLSR) between 2000 and 2015 were studied. IS was classified, based on the underlying mechanism, into large-artery atherosclerosis (LAA), cardio-embolism (CE), small-vessel occlusion (SVO), other determined aetiologies (OTH), and undetermined aetiologies (UND). After calculation of age-, sex-, and ethnicity-specific incidence rates by subtype for the 16-year period, we analysed trends using Cochran-Armitage tests, Poisson regression models, and locally estimated scatterplot smoothers (loess). A total of 3,088 patients with first IS were registered. Between 2000–2003 and 2012–2015, the age-adjusted incidence of IS decreased by 43% from 137.3 to 78.4/100,000/year (incidence rate ratio [IRR] 0.57, 95% CI 0.5–0.64). Significant declines were observed in all subtypes, particularly in SVO (37.4–18; *p* < 0.0001) and less in CE (39.3–25; *p* < 0.0001). Reductions were recorded in males and females, younger (<55 years old) and older (≥55 years old) individuals, and white and black ethnic groups, though not significantly in the latter (144.6–116.2; *p* = 0.31 for IS). A 4-fold increase in prior-to-stroke use of statins was found (adjusted odds ratio [OR] 4.39, 95% CI 3.29–5.86), and despite the increasing prevalence of hypertension (OR 1.54, 95% CI 1.21–1.96) and atrial fibrillation (OR 1.7, 95% CI 1.22–2.36), preventive use of antihypertensive and antiplatelet drugs was declining. A smaller number of participants in certain subgroup-specific analyses (e.g., black ethnicity and LAA subtype) could have limited the power to identify significant trends.

**Conclusions:**

The incidence of ISs has been declining since 2000 in all age groups but to a lesser extent in the black population. The reported changes in medication use are unlikely to fully explain the reduction in stroke incidence; however, innovative prevention strategies and better management of risk factors may contribute further reduction.

## Abstract 2

Hatem Wafa and colleagues use data from the South London Stroke Register to assess the contribution of prior risk factors, preventative treatments, and demographic disparities on stroke incidence during a 15-year period.

## Author summary

**Why was this study done?:**

- Worldwide, stroke is one of the leading causes of death and disability.
- The frequency of new incident cases has been declining over time in several parts of the world; nonetheless, recent studies in a number of countries reported an increase.
- Even with an overall decline in stroke incidence, an increase in one or more subtypes could be masked with a decrease in others.
- Monitoring trends of stroke incidence is critical to management of health services and defining priorities.

**What did the researchers do and find?:**

- We estimated the annual incidence rate of ischaemic stroke by different subtypes and age, sex, and ethnic groups and evaluated the trends in risk factors and medication use after accounting for the changes in population structure over time.
- An overall 43% risk reduction between 2000 and 2015 was estimated, which was mainly driven by a reduction in white groups and in strokes due to small-vessel disease.
- A concurrent increase in prior-to-stroke use of cholesterol-lowering agents was evident in all age, sex, and ethnic groups.

**What do these findings mean?:**

- The identified declining trends in ischaemic stroke incidence are unlikely to be solely due to the reported changes in medication use; however, better control of risk factors may bring further reduction.
- Estimates can be used by officials to plan future policy and to monitor the effect of new public health initiatives.
- Research should focus on developing better tools to identify individuals at risk.

## Introduction

Stroke is the second most common cause of death, accounting for 6.24 million deaths globally in 2015 [1]. It is also a leading cause of dependence and disability, ranked third worldwide [2]. In developed countries, most incident and prevalent cases are due to ischaemia, up to 87% and 81% respectively [3–5], and despite the reported decline in ischaemic stroke (IS) incidence [6–8], the absolute burden of the disease is expected to rise substantially as populations continue to grow and live longer [7].

Except for very few studies [9–12], population-based investigations of stroke incidence have largely been confined to pathological subtypes [6,13–16]. A more complex pattern of trends by IS aetiologies could have been masked, and characterising these trends can guide future prevention and therapeutic priorities. Although an overall risk reduction or stabilisation was observed in several parts of the world [6,11,12,17,18], Benatru and colleagues found an increase in the incidence of small-vessel occlusion (SVO) between 1985 and 2004, using data from the Dijon Register, France [9]. Similarly, studies from New Zealand [12] and Germany [11] have shown increases in the incidence of SVO during the 2002–2012 and 1995–2010 periods respectively, and an increase in large-artery atherosclerosis (LAA) has also been noted [12]. The incidence rate for cardio-embolism (CE) plateaued in Japan (1988–2004) [10], Germany (1995–2010) [11], and New Zealand (2002–2012) [12]. Most of these findings were coupled with unfavourable trends in cardiovascular health, particularly blood pressure, cholesterol levels, and diabetes.

Previous studies are limited by demography—having studied overwhelmingly white population [6,9,11]—or methodology—being reliant on only two separate time points for the estimation of temporal trends rather than continuous epidemiological monitoring [6,12]. Moreover, few have provided information on risk profiles in people with stroke [9,12]. We aimed to explore the trends in incidence and risk factor profile for IS aetiological subtypes over a 16-year period, using a community-based register of all cases from a defined multiethnic population of south London.

## Methods

### Procedures

We analysed data from the South London Stroke Register (SLSR), an ongoing observational study that since 1995 has conducted population-based case ascertainment of first-ever strokes in a defined population of inner London [19]. The study area comprises 22 electoral wards in the north of two London boroughs: Lambeth and Southwark [20]. According to the 2011 census data from the Office for National Statistics [21], the SLSR area was inhabited by 357,308 people, 56% of which were white, 25% black (14% black African, 7% black Caribbean, and 4% other black), and 18% of other ethnic backgrounds. Further details of the ethnic composition at different time periods are provided in Table A in S1 Appendix.

Surveillance methods have been described in detail elsewhere [19,22] and are summarised here. Patients were identified at five hospitals serving the study area—two within and three outside the study area. Additional community cases were identified by regular contact with all general practitioners (GPs) within the borders of the study area [23]. Standardised protocols were followed to ensure completeness of case ascertainment, which involves a multiple overlapping tracking system. Notification sources included the accident and emergency records, hospital wards, radiology records, death certificates, coroner’s records, hospital stroke registries, GP computer records, hospital medical staff, GPs and practice staff, community therapists, and bereavement officers [20]. Capture-recapture models estimated the completeness of case ascertainment in our population to be approximately 80% (between 75% and 88%), as shown in previous SLSR studies [18,19,24].

All data were collected prospectively by specially trained nurses, doctors, and fieldworkers who vouch for the completeness and accuracy of the data. Whenever possible, patients were assessed within 48 hours of referral to the SLSR, and data were checked against the patients’ GP and medical records [19]. Stroke diagnosis follows the World Health Organization criteria [25]. Pathological classification was based on neuroradiology (CT/MRI scans), CSF analysis, or autopsy results [18,19] and was further verified by a study clinician. Accordingly, patients were classified into cerebral infarction, primary intracerebral haemorrhage, or subarachnoid haemorrhage, whereas cases without pathological confirmation of subtype were undefined. Subtype classification of IS was carried out—using the Trial of ORG 10172 in Acute Stroke Treatment (TOAST) criteria [26]—into (1) LAA, (2) CE, (3) SVO, (4) other determined aetiologies (OTH), and (5) undetermined aetiologies (UND). The proportion of IS patients who received any brain scan increased from 95% in 2000–2003 to 100% in 2012–2015; MRI uptake increased from 14% to 35%.

Information collected at initial assessment included (1) demographic variables of age, sex, and self-definition of ethnic origin (1991 census question) [20], stratified into black (African, Caribbean, and other), white, and others (Asian, Pakistani, Indian, Bangladeshi, Chinese, and other); (2) premorbid risk factors of smoking (current versus quitter/never), alcohol intake (≥21 units/week for men, ≥14 units/week for women), hypertension (general practice or hospital records of systolic blood pressure > 140 mmHg or diastolic > 90 mmHg), diabetes mellitus, hypercholesterolemia (total cholesterol concentration ≥ 6 mmol/L), myocardial infarction, transient ischaemic attacks (TIAs), and atrial fibrillation (general practice or hospital records); and (3) premorbid prescription of antihypertensive drugs, antidiabetic medications (with oral hypoglycaemics or insulin), cholesterol-lowering agents, antiplatelets, and anticoagulants. Written informed consent and assent, when appropriate, were obtained from all participants or from a relative for the participants who were too impaired to provide written consent [19]. Ethical approval for the study was obtained from the ethics committees of Guy’s and St Thomas’ Hospital Trust, King’s College Hospital, Queens Square, and Westminster Hospital (London).

### Statistical analysis

Denominators used the intercensal population estimates for the SLSR area between 1991, 2001, and 2011 [21,27,28]. Age-, sex-, and ethnicity-specific proportions were applied to estimate the demographic composition of the study population between two adjacent censuses, assuming linear trends. The source population for the SLSR was extended to include other areas between 2004 and 2007, and estimations were based on the extended area population for this period. Incidence rates were calculated regardless of subsequent participation in the study as the number of the valid cases (with duplicates removed) divided by the number of residents in the SLSR area over the same period. The rates were calculated in the annual and quadrennial cohorts between Jan 1, 2000, and Dec 31, 2015, and were expressed per 100,000 persons per year. These were presented for the overall IS and the aetiological subtypes, each stratified by age (<55 years and ≥55 years), sex, and ethnicity. All incidences were age adjusted using direct methods to the 2011 census population of England and Wales and the 2013 European Standard Population; the latter is only presented in the Supporting information (Fig A, Fig B, and Table D in S1 Appendix). The 95% CIs were estimated assuming a Poisson distribution for the number of events. Time trends were analysed with the Cochran-Armitage tests, Poisson regression models, and locally estimated scatterplot smoothers (loess) [29]. Multiple logistic regressions were conducted to ascertain the changes in the premorbid variables across time, after adjusting for age, sex, and ethnicity. Time-by-ethnicity interaction term was also included in the models to account for the possible modification of the proportional effect of time on each risk factor by ethnicity. Backward elimination of the interaction term was performed if statistical significance of *p* < 0.05 was not reached.

To minimise analytic bias by missing data, multiple imputation with chained equations was applied to generate 20 datasets. Missing values for hypertension (1.3%), diabetes (1.6%), hypercholesterolemia (2.3%), atrial fibrillation (1.6%), myocardial infarction (2.3%), TIA (1.6%), drinking (9.8%), and smoking (7.8%) and for antihypertensive (2.5%), antidiabetic (0.9%), antiplatelet (13.5%), anticoagulant (8.5%), and cholesterol-lowering agents (6.3%) were imputed; each was modelled as a binomial using all variables in the study (including date of stroke onset). Parameter estimates were finally combined using Rubin’s principles [30]; these were very similar to the ones obtained from the nonimputed dataset. All analyses were performed with the statistical software R version 3.4.1.

## Results

### Study population

For the incident years in the 2000–2015 period, a total of 3,088 patients with IS were identified from a denominator of 5,959,964 person-years (an average of approximately 372,500 persons per year in the SLSR area). The distribution of these patients among the aetiological subtypes was as follows: LAA, 347 (11.2%); CE, 802 (26%); SVO, 785 (25.4%); OTH, 82 (2.7%); and UND, 1,072 (34.7%). The average age of IS onset was 70.7 years; 49.1% were females; 66.3% were white, 25.5% were black, and 8.3% were of other ethnic groups (Table 1). These characteristics varied significantly among different subtypes; CE had an older age of onset (74.4 years, *p* < 0.0001) and a higher proportion of females (54.4%, *p* < 0.001), whereas the frequency of black patients was the greatest in SVO (34.8%, *p* < 0.0001).

**Table 1:** Baseline characteristics of first-ever IS by aetiological subtypes.

	LAA (*n* = 347)	CE (*n* = 802)	SVO (*n* = 785)	OTH (*n* = 82)	UND (*n* = 1,072)	*p*-value
Age (mean [SD])	69.3 (14.3)	74.4 (14.1)	69.3 (13.5)	53.5 (16.7)	70.8 (14.4)	<0.0001*
Female	161 (46.4)	436 (54.4)	347 (44.2)	35 (42.7)	537 (50.1)	<0.001*
Year of stroke						<0.0001*
2000–2003	66 (19.0)	225 (28.1)	226 (28.8)	24 (29.3)	265 (24.7)	
2004–2007	132 (38.0)	275 (34.3)	266 (33.9)	37 (45.1)	407 (38.0)	
2008–2011	102 (29.4)	141 (17.6)	166 (21.1)	7 (8.5)	204 (19.0)	
2012–2015	47 (13.5)	161 (20.1)	127 (16.2)	14 (17.1)	196 (18.3)	
Ethnic group						<0.0001*
White	234 (67.4)	594 (74.1)	445 (56.7)	49 (59.8)	725 (67.6)	
Black	88 (25.4)	147 (18.3)	273 (34.8)	26 (31.7)	252 (23.5)	
Other/unknown	25 (7.2)	61 (7.6)	67 (8.5)	7 (8.5)	95 (8.9)	

Data are count (%) unless otherwise indicated.

* denotes significant difference among subtypes (*p* < 0.05). *p*-Values were obtained from ANOVA or chi-squared tests as appropriate.

Abbreviations: CE, cardio-embolism; IS, ischaemic stroke; LAA, large-artery atherosclerosis; OTH, other determined aetiologies; SVO, small-vessel occlusion; UND, undetermined aetiologies.

Trends in baseline characteristics over time are summarised in Table 2. Over the study period, the average age of IS onset has significantly decreased by three years (72.3 in 2000–2003 compared with 69.3 in 2012–2015, *p* < 0.001), and the distribution of IS patients among different ethnic groups has also changed, with a declining proportion of white patients and a rising contribution of black patients (*p* < 0.0001). However, parallel changes in the underlying structure of the at-risk population were observed (Table A in S1 Appendix). In the surveillance network, the proportion of residents aged ≥65 years decreased from approximately 9% in 2000–2003 to 6.6% in 2012–2015 (*p* < 0.0001). Furthermore, the proportions of female, white, and black residents have declined (*p* < 0.0001).

**Table 2:** Changes in baseline characteristic of first-ever IS over time.

	2000–2003 (*n* = 806)	2004–2007 (*n* = 1,117)	2008–2011 (*n* = 620)	2012–2015 (*n* = 545)	*p*-value
Age (mean [SD])	72.31 (13.21)	70.90 (14.27)	69.57 (15.84)	69.29 (15.31)	<0.001*
Age >55 years	725 (90.0)	960 (85.9)	494 (79.7)	450 (82.6)	<0.0001*
Female	426 (52.9)	524 (46.9)	316 (51.0)	250 (45.9)	0.02*
Ethnicity					<0.0001*
White	589 (73.1)	769 (68.8)	381 (61.5)	308 (56.5)	
Black	150 (18.6)	262 (23.5)	184 (29.7)	190 (34.9)	
Other/unknown	67 (8.3)	86 (7.7)	55 (8.9)	47 (8.6)	

Data are count (%) unless otherwise indicated. *p*-Values were obtained from the Cochran-Armitage tests for trend.

* denotes significance (*p* < 0.05).

Abbreviation: IS, ischaemic stroke.

### Trends in IS incidence

Numerator/denominator data and the crude incidence rates for all ISs (stratified by age, sex, and ethnicity) are provided in Table B in S1 Appendix. The crude annual incidence per 100,000 population for any first-ever IS was 73.5 (95% CI 68.5–78.7) in 2000–2003 compared to 36.8 (95% CI 33.8–40) in 2012–2015. Similar reductions were observed in all aetiological subtypes, but variations existed among different strata of age, sex, and ethnicity.

**Fig 1:**
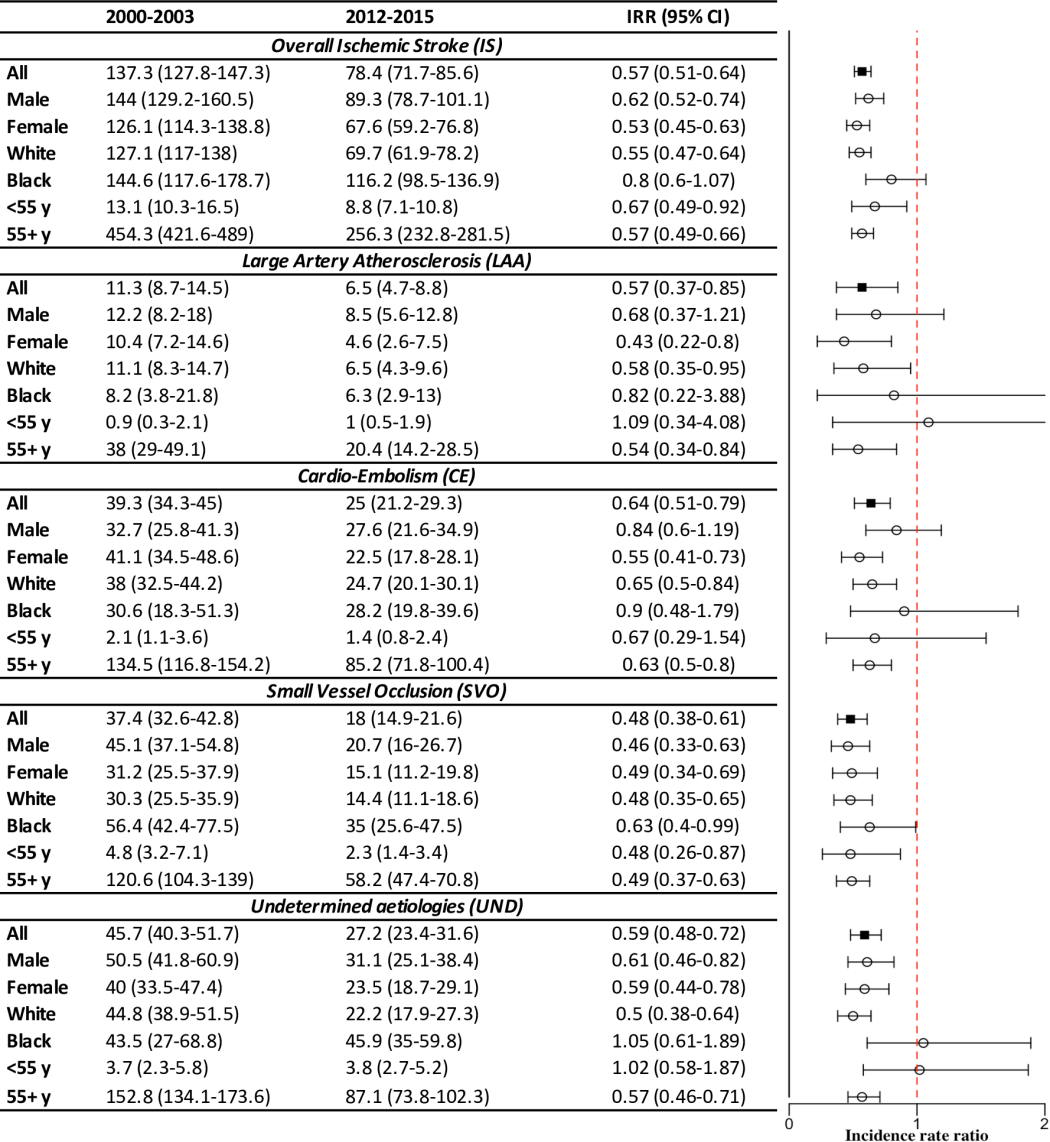
Standardised† annual incidences per 100,000 per year (95% CI) of first ISs over time, stratified by sex, ethnicity, and age groups. † To the 2011 census population of England and Wales. Complete information for other periods is available in Table C in S1 Appendix. IRR, incidence rate ratio; IS, ischaemic stroke.

**Fig 2:**
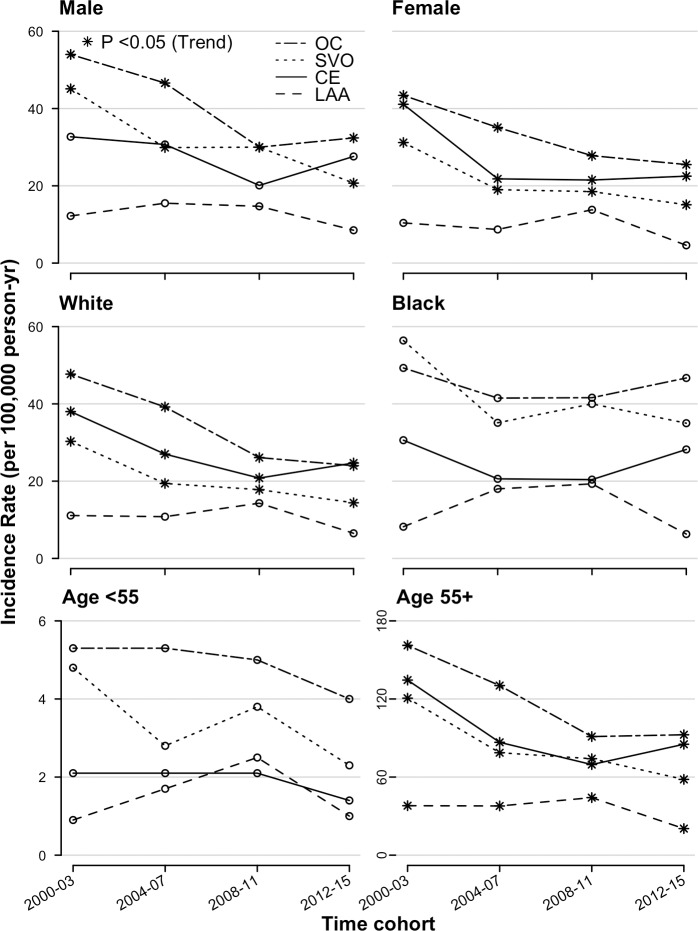
Trends in the age-standardised† annual incidence per 100,000 per year for first-ever ISs by sex, ethnicity, and age. † To the 2011 population of England and Wales. *p*-Values were obtained from the Cochran-Armitage tests for trend. * denotes significant trends (*p* < 0.05). CE, cardio-embolism; IS, ischaemic stroke; LAA, large-artery atherosclerosis; OC, other causes; SVO, small-vessel occlusion.

**Fig 3:**
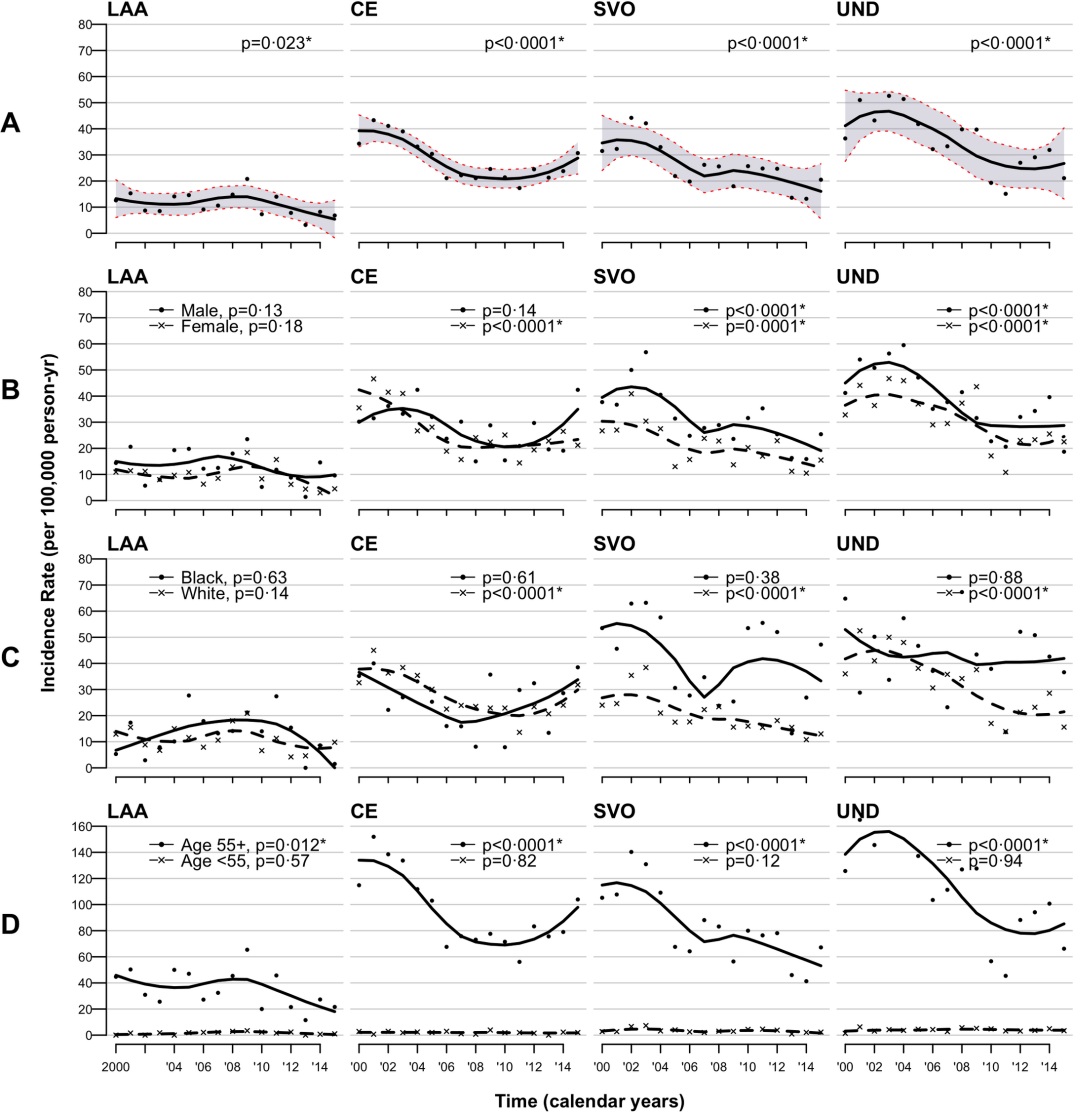
**Trends in standardised† incidences per 100,000 per calendar year for IS aetiological subtypes; (A) overall, (B) by sex, (C) by ethnicity, and (D) by age groups**. † To the 2011 census population of England and Wales. Data are the observed values with regression fitted lines (loess). *p*-Values were obtained from the Cochran-Armitage tests for trend. * denotes significant trends (*p* < 0.05). CE, cardio-embolism; IS, ischaemic stroke; LAA, large-artery atherosclerosis; loess, locally estimated scatterplot smoothers; SVO, small-vessel occlusion; UND, undetermined aetiologies.

Rates age adjusted to the 2011 census population of England and Wales are shown in Fig 1 and Fig 2. Fig 3 further portrays the data annually for aetiological subtypes with regression-fitted lines; figures adjusted to the 2013 European Standard Population showed similar trends and are included in the Supporting information (Table D, Fig A, and Fig B in S1 Appendix). Between 2000–2003 and 2012–2015, there was an overall 43% reduction in the incidence of first-ever IS from 137.3 to 78.4 per 100,000 persons per year (incidence rate ratio [IRR] 0.57, 95% CI 0.5–0.64), which was mainly driven by reductions in SVO (37.4–18, *p* < 0.0001), CE (39.3–25, *p* < 0.0001), and finally LAA (11.3–6.5, *p* = 0.039).

Females had not only greater reductions of IS incidence than males (47% versus 38% respectively) but also lower risks at all time points (Fig 1). This disparity is due to a sizeable decline in the incidence of CE stroke among females (Fig 2 and Fig 3), a 45% reduction (*p* < 0.0001) compared with 16% in males (*p* = 0.09). As for ethnic differences, significant declines were seen in the white population (IRR 0.55, 95% CI 0.47–0.64), and an overall 20% risk reduction was observed in the black population (IRR 0.8, 95% CI 0.6–1.07), which was only significant in SVO (IRR 0.63, 95% CI 0.4–0.99). The decline in SVO among black people was remarkable until 2006, which had no identified trend (Fig 2 and Fig 3). In terms of IS aetiologies, there were major reductions in the age-standardised risk of SVO (IRR 0.48, 95% CI 0.38–0.61), LAA (IRR 0.57, 95% CI 0.37–0.85), and finally CE (IRR 0.64, 95% CI 0.51–0.79) between 2000–2003 and 2012–2015. These were confined to females, white groups, and the elderly ≥55 years old for both LAA and CE besides males and blacks for SVO (Fig 1). However, trends were not identified in either females and whites for LAA or blacks and young people for SVO (Fig 3).

### Trends in risk factor and medication use

**Fig 4:**
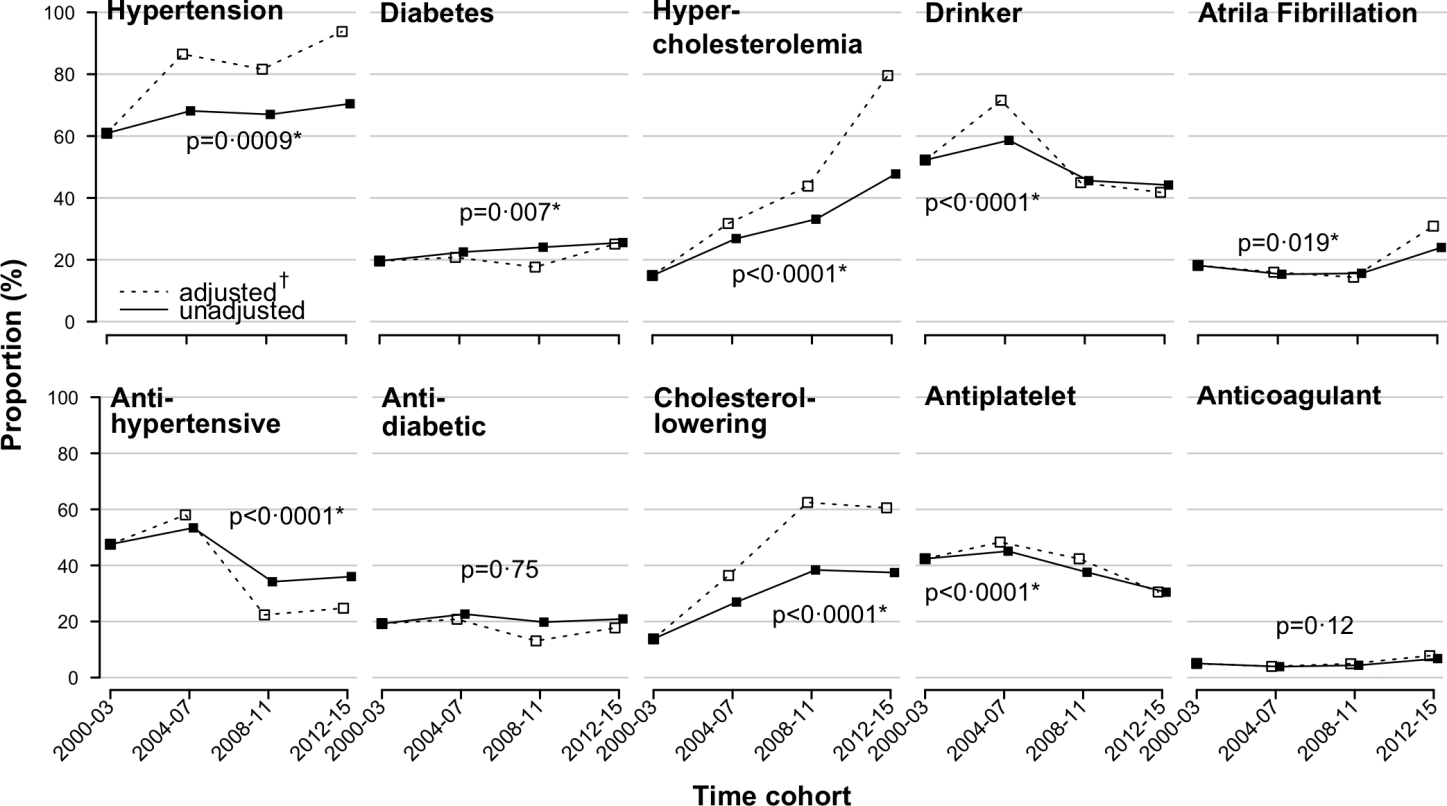
Prior risk factors and medication use over time in patients with incident first-ever IS. *p*-Values were obtained by the Cochran-Armitage tests for trend and are presented for the unadjusted rates. * denotes significant trends (*p* < 0.05). † adjusted for age, sex, and ethnicity, allowing for interaction between time and ethnicity as appropriate. IS, ischaemic stroke.

**Fig 5:**
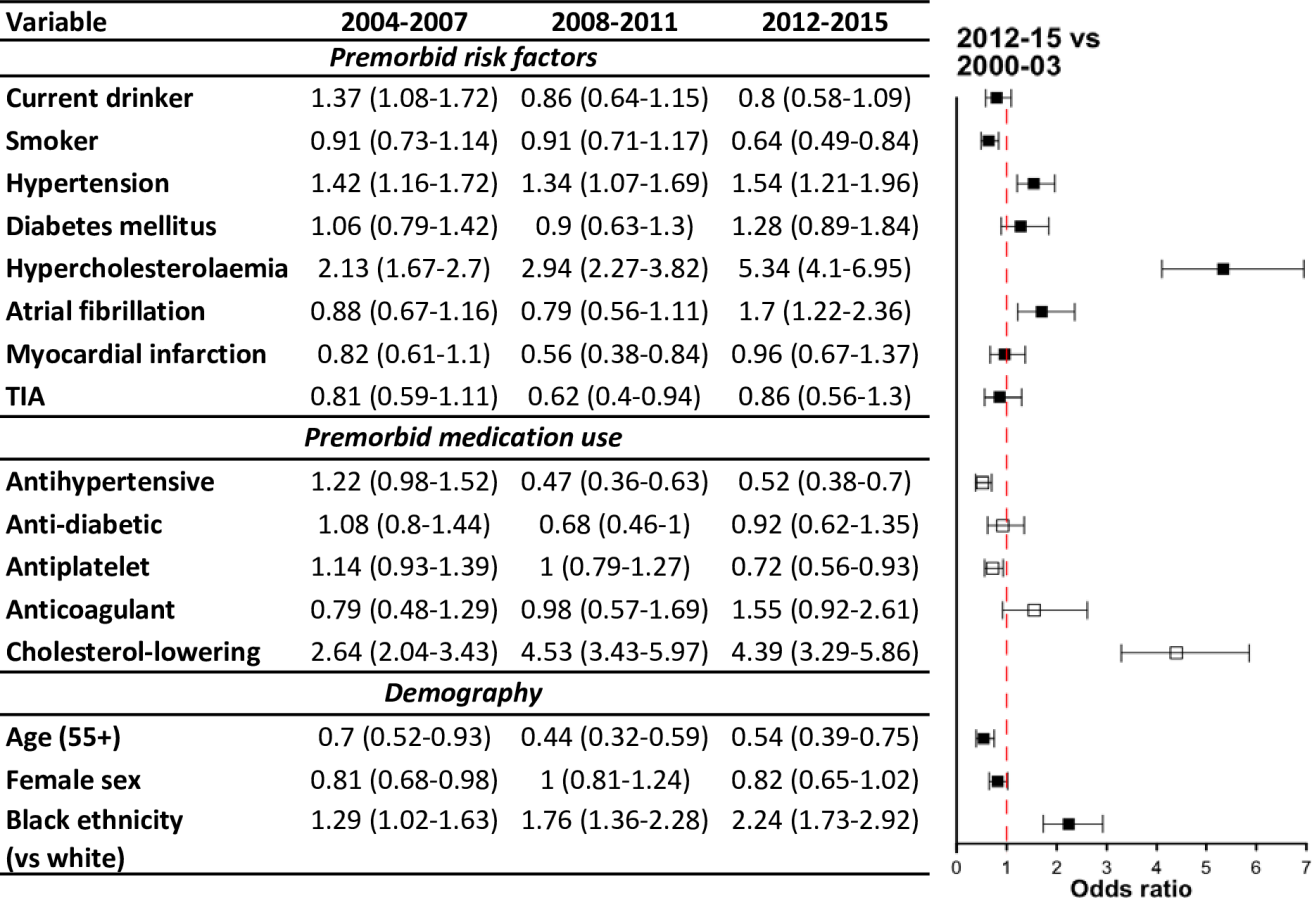
Multiply adjusted† changes in risk factor profile in first-ever IS patients (with reference to 2000–2003 index cases). Data are OR (95% CI). † for age, sex, ethnicity, and possible interaction between time and ethnicity as appropriate, after multiple imputation of missing values. IS, ischaemic stroke; OR, odds ratio; TIA, transient ischaemic attack.

Fig 4 shows the levels and trends of selected prestroke variables among first-ever IS patients (unadjusted and adjusted to demographic changes). The magnitude of change during every time period with reference to 2000–2003 is shown in Fig 5, as obtained from multiply adjusted models for age, sex, ethnicity, and possible time-by-ethnicity interaction. Compared with 2000–2003, 20% fewer alcohol drinkers were identified in the 2012–2015 cohort (adjusted odds ratio [OR] 0.8, 95% CI 0.58–1.09). The proportion of patients who were regular or ex-smokers decreased significantly (OR 0.64, 95% CI, 0.49–0.84). There were, however, surprising trends, with increasing prevalence of premorbid hypertension (*p* < 0.001), hypercholesterolemia (*p* < 0.0001), and atrial fibrillation (*p* = 0.019). The independent increases in these risk factors between 2000–2003 and 2012–2015 were estimated at 54%, 434%, and 70% respectively (Fig 5). Moreover, preventive use of medications fell, except for antidiabetic medications, anticoagulants, and cholesterol-lowering agents. The odds of premorbid use of cholesterol-lowering drugs have quadrupled during the study period, after adjusting for demographic variations (OR 4.39, 95% CI 3.29–5.86).

Similar profiles were identified for different demographic and aetiological subgroups (Tables E-N and Figs C-L in S1 Appendix). Increasing rates of atrial fibrillation (*p* = 0.013) were identified in males, whereas hypertension increased in females only (*p* < 0.0001). The black population had consistently higher levels of hypertension and diabetes across all time points. In addition, an increasing trend of atrial fibrillation was identified in black groups (*p* < 0.001) but not the white (*p* = 0.12). Smoking and drinking were more prevalent in the younger patients compared to those aged ≥55 years, and irrespective of demographic variations, hypertension increased in the former by 98% and in the latter by 55%.

## Discussion

The community-based data from a large and diverse population of south London show a significant decrease in the annual incidence of first-ever ISs in the 2000–2015 period. The reductions were unequally shared between different demographic subgroups; no significant declines were found in the black population (only a modest decrease in SVO). These findings were despite an increased frequency of most cardiovascular risk factors for IS, except for a trend toward lower tobacco smoking and alcohol consumption.

Few studies can accurately track the incidence of ISs over time, and our study provides robust evidence that indicates a decline over time. Data from the Oxford Vascular Study (OXVASC) showed a 27% decline in the age-standardised incidence of IS between 1983–1984 and 2002–2004 in a predominantly white population (94%) [6]. In the SLSR white group, a greater reduction (45%) was detected over a shorter, and more recent, period of time (2000–2015). Indeed, improvements in public awareness, health, and healthcare might have played a role. However, our SLSR area was undergoing rapid economic development over the past decades, and a subsequent greater influx of migrants, who are arguably healthier, might partly account for this disparity [18,31,32]. Unlike OXVASC, we explored trends on the basis of continuous epidemiological surveillance rather than solely relying on two separate time points. Furthermore, lack of ethnic diversity in OXVASC limits the generalisability of the results to other settings of different ethnic makeup, and finally, the small sample size in OXVASC (*n* = 223 versus 3,088 in our study) did not permit enough power to characterise the differences among IS aetiologies.

In contrast to studies in France [9], Germany [11], and New Zealand [12] that suggested an increase in SVO, we observed a progressive decline in the incidence of SVO over time. In these studies, it is difficult to distinguish a true rise from one falsely resulting from expanded use of neuroimaging techniques, which are also more sensitive (e.g., MRI). The improved diagnostic accuracy would presumably have a larger impact on SVO identification because of the milder nature of the injury. The result would be improved ascertainment and an apparent increase in incidence in the later years compared to the earlier ones. Hence, our observed trends in the incidence of SVO since 2000 are even conservative.

The Auckland Regional Community Stroke Studies suggested no change in CE incidence by comparing two separate years only (2002/2003 and 2011/2012) [12]. In contrast, our study shows an overall 36% incidence reduction in CE stroke from 2000 to 2015. The reduction, however, was more pronounced during the earlier years of the study, and a stable, or even increasing, trend was observed in the more recent years (Fig 3). This might indicate improved recognition of CE due to increased use of cardiac monitoring techniques—use of ECG increased from about 90% in 2000/2003 to almost 100% in 2012/2015. It might as well be explained by, for example, the increase in atrial fibrillation (70%) (Fig 5)—a finding that was similarly reported in other settings [12]. Although such a finding is alarming, it represents a potential opportunity for prevention and better risk management, particularly that inadequate changes in the use of atrial fibrillation–related medications were also identified.

In consensus with previous literature [32–34], the black population in our study was at increased risk of IS compared with the white group. The greatest gap existed in the SVO subtype, in which consistently higher and relatively static incidence over time was found in the black population compared to those of white ethnic origin (Fig 3). In association, higher levels of hypertension and diabetes were recorded in black patients at all time periods, which may, at least partially, account for the slower reduction in the incidence of ISs and SVO in particular. This is because (1) the association of these risk factors with SVO has been previously established [35,36], (2) an earlier onset of vascular risk factors among the black population is well documented [37], and (3) these conditions are more likely to be resistant to treatments in the black ethnic groups [38].

A trend toward an earlier age of stroke onset is evident in the United States [39,40]. However, despite the significant decrease in the age of IS onset in our population (Table 2), that trend is likely to be artificial for two reasons. First, there were no observed inclines in the age-standardised incidence of IS among young people aged <55 years (Fig 2). Second, the proportion of older residents ≥65 years of age in our at-risk population of 2000–2003 was 26% larger than that in the later 2012–2015 period (Table A in S1 Appendix). This higher proportion will eventually contribute more patients over 65 years of age to the corresponding temporal cohort of patients, which would misleadingly suggest a trend toward a younger onset of stroke if not taken into account. In Dijon, for example, an increase in the age of stroke onset by approximately 6 years was noted, yet a 51% increase in the proportion of old residents (>85 years of age) was also found, which might be responsible [9]. As such, any interpretation of a changing age of onset over time should be made in the context of demographic variations in the source population over the same time frame; otherwise, incorrect conclusions may be drawn.

In our investigation of risk factor profile, a worsening trend was observed in most indicators of cardiovascular health, except for smoking and drinking. These findings are in line with national [41] and global [42–44] statistics. The use of cholesterol-lowering agents has quadrupled since 2000 in our population, which was consistently seen in all aetiologic and demographic subgroups. There is a strong evidence base for the role of statins in preventing strokes and for their association with an approximately 20% risk reduction [45–47]. Therefore, the declining incidence in our study might be largely attributable to the observed increase in statin use. However, the magnitude of reduction in stroke risk (43%) suggests involvement of other unidentified factors. Factors that govern IS incidence are complex and overlapping, and our observed trends in the prevalence of risk factors and medication use cannot easily explain the observed reduction in incidence. There are several other considerations we could not adjust for, including but not limited to physical activity, psychosocial stress, medication adherence, and other environmental, behavioural, and genetic factors [48–51]. The 2016 Health Survey for England, for instance, suggested increasing proportions of adults meeting recommendations for levels of physical activity and an improvement in fruit and vegetable consumption [41]. In addition, numerous United Kingdom–based investigations have shown a decreasing prevalence of resistant hypertension [41,52,53]. The collective effect is associated with a lower incidence of ISs. It is therefore possible that improvements in these factors have offset the anticipated harm from the worsening trends in risk factors’ prevalence in our study.

This is the largest population-based study that describes the changes in IS incidence and risk factors by TOAST aetiological subtypes with further investigation by age, sex, and ethnic groups. Our notification system is as consistent and accurate as possible, but we acknowledge that the completeness of case ascertainment has increased over years (75% in 2001–2002 to 88% in 2005–2006) [18,19]. Although this increase is not significant, any resulting bias is likely to increase the sensitivity for finding IS cases in recent years and should create bias against finding a downward trend. Another strength of our study is the long period of continuous identification of people with stroke in a multiethnic area of south London; the sample size accrued over a period of 16 years provided sufficient power to examine long-term trends in aetiological and demographic subgroups. Nevertheless, statistical power was limited in certain subgroup-specific analyses because of low numbers—in LAA subgroups and young people. One limitation of this study is our inability to explore the effects of some putative risk factors for IS as possible explanations for the observed trends. In addition, we were unable to confirm changes in the severity of the investigated risk factor because of lack of data and unavailability of the measures on continuous scales. We also acknowledge that the frequency of missing values in IS risk factors was greater in the earlier years compared to the later ones, which might have distorted the trends in risk factors over time. On average, data were unavailable in 4.5% of the investigated predictors with a range of 0.9%–13.5%. However, nonresponse was plausibly random and displayed no systematic pattern, and multiple imputation analysis was performed to minimise any resulting bias. Finally, inherent limitations of using TOAST classification need to be addressed, such as the high proportion of cases with UND due to missing new pathophysiological and diagnostic knowledge.

In conclusion, disproportionate reductions in the incidence of ISs have been identified in London, UK, across different demographic groups. Our study provides cautious hope that prevention, or at least delaying the onset, of some strokes is possible through better management of risk factors. It also emphasises the importance of targeted prevention strategies to enhance the current trends and avert future inclines.

## Supporting information

S1 STROBE Checklist (PDF)

S1 Appendix (DOCX)

S1 Text Analysis plan. (DOCX)

## Abbreviations

CE: cardio-embolism
GP: general practitioner
IRR: incidence rate ratio
IS: ischaemic stroke
LAA: large-artery atherosclerosis
loess: locally estimated scatterplot smoothers
OC: other causes
OR: odds ratio
OTH: other determined aetiologies
OXVASC: Oxford Vascular Study
SLSR: South London Stroke Register
SVO: small-vessel occlusion
TIA: transient ischaemic attack
TOAST: Trial of ORG 10172 in Acute Stroke Treatment
UND: undetermined aetiologies
