# Supplementary material for: Long-term trends in incidence and risk factors for ischaemic stroke subtypes: Prospective population study of the South London Stroke Register
Source: PLoS Med. 2018 Oct 5;15(10):e1002669. doi: 10.1371/journal.pmed.1002669 (PMC6173399; doi:10.1371/journal.pmed.1002669)
Supplement: S1 Appendix — (DOCX) [file pmed.1002669.s002.docx]

**Supplementary Appendix**

**Long-Term Trends in Incidence and Risk Factors for Ischaemic Stroke Subtypes: Prospective Population Study of The South London Stroke Register**

Hatem A Wafa, MPH; Charles D.A. Wolfe, FFPH; Anthony Rudd, FRCP; Yanzhong Wang, PhD

# Contents

[1 Demographic changes in the source population 4](#_Toc520464873)

[2 Crude incidence rates 5](#_Toc520464874)

[3 Standardised incidence rates 9](#_Toc520464875)

[3.1 To census population of England & Wales (2011) – EW11 9](#_Toc520464876)

[3.2 To European Standard Population (2013) – ESP13 10](#_Toc520464877)

[4 Trends in risk factors in male patients 14](#_Toc520464878)

[5 Trends in risk factors in female patients 15](#_Toc520464879)

[6 Trends in risk factors in white patients 16](#_Toc520464880)

[7 Trends in risk factors in black patients 17](#_Toc520464881)

[8 Trends in risk factors in patients <55 years 18](#_Toc520464882)

[9 Trends in risk factors in patients 55+ years 19](#_Toc520464883)

[10 Trends in risk factors in patients with LAA 20](#_Toc520464884)

[11 Trends in risk factors in patients with CE 21](#_Toc520464885)

[12 Trends in risk factors in patients with SVO 22](#_Toc520464886)

[13 Trends in risk factors in patients with UND 23](#_Toc520464887)

# List of tables

[**Table A:** Demographic changes in SLSR source population 4](#_Toc520465152)

[**Table B:** Crude annual incidence of ischaemic strokes over time 5](#_Toc520465153)

[**Table C:** Age-adjusted trends in annual incidence ̶ EW11 9](#_Toc520465154)

[**Table D:** Age-adjusted trends in annual incidence ̶ ESP13 10](#_Toc520465155)

[**Table E:** Premorbid risk factors and medication use in males 14](#_Toc520465156)

[**Table F:** Premorbid risk factors and medication use in females 15](#_Toc520465157)

[**Table G:** Premorbid risk factors and medication use in white patients 16](#_Toc520465158)

[**Table H:** Premorbid risk factors and medication use in black patients 17](#_Toc520465159)

[**Table I:** Premorbid risk factors and medication use in patients <55y 18](#_Toc520465160)

[**Table J:** Premorbid risk factors and medication use in patients 55+ 19](#_Toc520465161)

[**Table K:** Premorbid risk factors and medication use in LAA stroke 20](#_Toc520465162)

[**Table L:** Premorbid risk factors and medication use in CE stroke 21](#_Toc520465163)

[**Table M:** Premorbid risk factors and medication use in SVO stroke 22](#_Toc520465164)

[**Table N:** Premorbid risk factors and medication use in UND stroke 23](#_Toc520465165)

# List of Figures

[**Fig A:** Trends in age-adjusted incidence by demographic groups ̶ ESP13 11](#_Toc520459940)

[**Fig B:** Trends in age-adjusted incidences aetiological subtypes ̶ ESP13 13](#_Toc520459941)

[**Fig C:** Prior risk factors and medication use over time in males 14](#_Toc520459942)

[**Fig D:** Prior risk factors and medication use over time in females 15](#_Toc520459943)

[**Fig E:** Prior risk factors and medication use over time in white patients 16](#_Toc520459944)

[**Fig F:** Prior risk factors and medication use over time in black patients 17](#_Toc520459945)

[**Fig G:** Prior risk factors and medication use over time in patients <55y 18](#_Toc520459946)

[**Fig H:** Prior risk factors and medication use over time in patients 55+ 19](#_Toc520459947)

[**Fig I:** Prior risk factors and medication use over time in LAA patients 20](#_Toc520459948)

[**Fig J:** Prior risk factors and medication use over time in CE patients 21](#_Toc520459949)

[**Fig K:** Prior risk factors and medication use over time in SVO patients 22](#_Toc520459950)

[**Fig L:** Prior risk factors and medication use over time in UND patinets 23](#_Toc520459951)

# Demographic changes in the source population

**Table A: Changes in demographic composition of the SLSR population over time.**

|  | **2000-2003** | **2004-2007** | **2008-2011** | **20121-2015** | **P-value** |
| --- | --- | --- | --- | --- | --- |
|  | **(n=1097284)** | **(n=1974988)** | **(n=1405668)** | **(n=1482024)** |  |
| **Age** |  |  |  |  | <0.0001* |
| <45 | 833532 (76) | 1459428 (73.9) | 1051112 (74.8) | 1104616 (74.5) |  |
| 45-54 | 98948 (9) | 214980 (10.9) | 161384 (11.5) | 184024 (12.4) |  |
| 55-64 | 67260 (6.1) | 131248 (6.6) | 90556 (6.4) | 96284 (6.5) |  |
| 65-74 | 53156 (4.8) | 91556 (4.6) | 55956 (4) | 52832 (3.6) |  |
| 75-84 | 33956 (3.1) | 57900 (2.9) | 34492 (2.5) | 32164 (2.2) |  |
| 85+ | 10432 (1) | 19876 (1) | 12168 (0.9) | 12104 (0.8) |  |
| **Age 65+ y** | 97544 (8.9) | 169332 (8.6) | 102616 (7.3) | 97100 (6.6) | <0.0001* |
| **Female** | 549452 (50.1) | 997184 (50.5) | 698800 (49.7) | 733220 (49.5) | <0.0001* |
| **Ethnicity** |  |  |  |  | <0.0001* |
| White | 683076 (62.3) | 1165900 (59) | 800220 (56.9) | 811064 (54.7) |  |
| Black | 315512 (28.8) | 526272 (26.6) | 362668 (25.8) | 366448 (24.7) |  |
| Other/unknown | 98696 (9) | 282816 (14.3) | 242780 (17.3) | 304512 (20.5) |  |

Data are count (%). P-values were obtained from the Cochran-Armitage tests for trend. * denotes significance (p<0.05).

# Crude incidence rates

**Table B:** **Crude annual incidences per 100,000 per year (95% CI) of first ischaemic stroke over time, stratified by stroke subtypes and age, sex, and ethnicity.**

|  | **2000-2003** | | **2004-2007** | | **2008-2011** | | **2012-2015** | | **P-value** | **IRR (95% CI) (2012-15 vs 2000-03)** |  |
| --- | --- | --- | --- | --- | --- | --- | --- | --- | --- | --- | --- |
|  | **n / N** | **Rate (95% CI)** | **n / N** | **Rate (95% CI)** | **n / N** | **Rate (95% CI)** | **n / N** | **Rate (95% CI)** |  |  |  |
| ***Ischaemic Stroke (IS)*** | | | | | | | | | | | |
| **All** | 806/1097284 | 73.5 (68.5-78.7) | 1117/1974988 | 56.6 (53.3-60) | 620/1405668 | 44.1 (40.7-47.7) | 545/1482024 | 36.8 (33.8-40) | <0.0001* | 0.5 (0.45-0.56) |  |
| **Sex** |  |  |  |  |  |  |  |  |  |  |  |
| Male | 380/547832 | 69.4 (62.6-76.7) | 593/977804 | 60.6 (55.9-65.7) | 304/706868 | 43 (38.3-48.1) | 295/748804 | 39.4 (35-44.2) | <0.0001* | 0.57 (0.49-0.66) |  |
| Female | 426/549452 | 77.5 (70.3-85.3) | 524/997184 | 52.5 (48.1-57.2) | 316/698800 | 45.2 (40.4-50.5) | 250/733220 | 34.1 (30-38.6) | <0.0001* | 0.44 (0.38-0.51) |  |
| **Ethnicity** |  |  |  |  |  |  |  |  |  |  |  |
| White | 589/683076 | 86.2 (79.4-93.5) | 769/1165900 | 66 (61.4-70.8) | 381/800220 | 47.6 (43-52.6) | 308/811064 | 38 (33.9-42.5) | <0.0001* | 0.44 (0.38-0.51) |  |
| Black | 150/315512 | 47.5 (40.2-55.8) | 262/526272 | 49.8 (43.9-56.2) | 184/362668 | 50.7 (43.7-58.6) | 190/366448 | 51.8 (44.7-59.8) | 1 | 1.09 (0.88-1.35) |  |
| **Age groups** |  |  |  |  |  |  |  |  |  |  |  |
| <55 y | 81/932480 | 8.7 (6.9-10.8) | 157/1674408 | 9.4 (8-11) | 126/1212496 | 10.4 (8.7-12.4) | 95/1288640 | 7.4 (6-9) | 1 | 0.85 (0.63-1.14) |  |
| 55+ y | 725/164804 | 439.9 (408.5-473.1) | 960/300580 | 319.4 (299.5-340.2) | 494/193172 | 255.7 (233.7-279.3) | 450/193384 | 232.7 (211.7-255.2) | <0.0001* | 0.53 (0.47-0.59) |  |
| **Age** |  |  |  |  |  |  |  |  |  |  |  |
| <45 | 28/833532 | 3.4 (2.2-4.9) | 62/1459428 | 4.2 (3.3-5.4) | 44/1051112 | 4.2 (3-5.6) | 38/1104616 | 3.4 (2.4-4.7) | 1 | 1.02 (0.63-1.68) |  |
| 45-54 | 53/98948 | 53.6 (40.1-70.1) | 95/214980 | 44.2 (35.8-54) | 82/161384 | 50.8 (40.4-63.1) | 57/184024 | 31 (23.5-40.1) | 0.07 | 0.58 (0.4-0.84) |  |
| 55-64 | 129/67260 | 191.8 (160.1-227.9) | 181/131248 | 137.9 (118.5-159.5) | 93/90556 | 102.7 (82.9-125.8) | 112/96284 | 116.3 (95.8-140) | 0.0001* | 0.61 (0.47-0.78) |  |
| 65-74 | 219/53156 | 412 (359.2-470.3) | 282/91556 | 308 (273.1-346.1) | 128/55956 | 228.8 (190.8-272) | 111/52832 | 210.1 (172.8-253) | <0.0001* | 0.51 (0.4-0.64) |  |
| 75-84 | 237/33956 | 698 (611.9-792.7) | 327/57900 | 564.8 (505.2-629.4) | 166/34492 | 481.3 (410.8-560.3) | 139/32164 | 432.2 (363.3-510.3) | <0.0001* | 0.62 (0.5-0.76) |  |
| 85+ | 140/10432 | 1342 (1128.9-1583.6) | 170/19876 | 855.3 (731.6-994) | 107/12168 | 879.4 (720.7-1062.6) | 88/12104 | 727 (583.1-895.7) | 0.0002* | 0.54 (0.41-0.71) |  |
| ***Large Artery Atherosclerosis (LAA)*** | | | | | | | | | | | |
| **All** | 66/1097284 | 6 (4.7-7.7) | 132/1974988 | 6.7 (5.6-7.9) | 102/1405668 | 7.3 (5.9-8.8) | 47/1482024 | 3.2 (2.3-4.2) | 0.012* | 0.53 (0.36-0.76) |  |
| **Sex** |  |  |  |  |  |  |  |  |  |  |  |
| Male | 32/547832 | 5.8 (4-8.2) | 77/977804 | 7.9 (6.2-9.8) | 47/706868 | 6.6 (4.9-8.8) | 30/748804 | 4 (2.7-5.7) | 0.29 | 0.69 (0.42-1.13) |  |
| Female | 34/549452 | 6.2 (4.3-8.6) | 55/997184 | 5.5 (4.2-7.2) | 55/698800 | 7.9 (5.9-10.2) | 17/733220 | 2.3 (1.4-3.7) | 0.09 | 0.37 (0.2-0.66) |  |
| **Ethnicity** |  |  |  |  |  |  |  |  |  |  |  |
| White | 51/683076 | 7.5 (5.6-9.8) | 85/1165900 | 7.3 (5.8-9) | 69/800220 | 8.6 (6.7-10.9) | 29/811064 | 3.6 (2.4-5.1) | 0.06 | 0.48 (0.3-0.75) |  |
| Black | 10/315512 | 3.2 (1.5-5.8) | 37/526272 | 7 (5-9.7) | 28/362668 | 7.7 (5.1-11.2) | 13/366448 | 3.5 (1.9-6.1) | 1 | 1.12 (0.49-2.62) |  |
| **Age groups** |  |  |  |  |  |  |  |  |  |  |  |
| <55 y | 5/932480 | 0.5 (0.2-1.3) | 21/1674408 | 1.3 (0.8-1.9) | 24/1212496 | 2 (1.3-2.9) | 11/1288640 | 0.9 (0.4-1.5) | 1 | 1.59 (0.58-5.05) |  |
| 55+ y | 61/164804 | 37 (28.3-47.5) | 111/300580 | 36.9 (30.4-44.5) | 78/193172 | 40.4 (31.9-50.4) | 36/193384 | 18.6 (13-25.8) | 0.026* | 0.5 (0.33-0.75) |  |
| **Age** |  |  |  |  |  |  |  |  |  |  |  |
| <45 | 1/833532 | 0.1 (0-0.7) | 5/1459428 | 0.3 (0.1-0.8) | 10/1051112 | 1 (0.5-1.7) | 4/1104616 | 0.4 (0.1-0.9) | 1 | 3.02 (0.45-59.05) |  |
| 45-54 | 4/98948 | 4 (1.1-10.4) | 16/214980 | 7.4 (4.3-12.1) | 14/161384 | 8.7 (4.7-14.6) | 7/184024 | 3.8 (1.5-7.8) | 1 | 0.94 (0.28-3.59) |  |
| 55-64 | 12/67260 | 17.8 (9.2-31.2) | 23/131248 | 17.5 (11.1-26.3) | 15/90556 | 16.6 (9.3-27.3) | 7/96284 | 7.3 (2.9-15) | 0.35 | 0.41 (0.15-1.01) |  |
| 65-74 | 18/53156 | 33.9 (20.1-53.5) | 39/91556 | 42.6 (30.3-58.2) | 19/55956 | 34 (20.4-53) | 12/52832 | 22.7 (11.7-39.7) | 1 | 0.67 (0.31-1.38) |  |
| 75-84 | 20/33956 | 58.9 (36-91) | 35/57900 | 60.4 (42.1-84.1) | 27/34492 | 78.3 (51.6-113.9) | 11/32164 | 34.2 (17.1-61.2) | 1 | 0.58 (0.27-1.19) |  |
| 85+ | 11/10432 | 105.4 (52.6-188.7) | 14/19876 | 70.4 (38.5-118.2) | 17/12168 | 139.7 (81.4-223.7) | 6/12104 | 49.6 (18.2-107.9) | 1 | 0.47 (0.16-1.24) |  |
| ***Cardio-Embolism (CE)*** | | | | | | | | | | | |
| **All** | 225/1097284 | 20.5 (17.9-23.4) | 275/1974988 | 13.9 (12.3-15.7) | 141/1405668 | 10 (8.4-11.8) | 161/1482024 | 10.9 (9.3-12.7) | <0.0001* | 0.53 (0.43-0.65) |  |
| **Sex** |  |  |  |  |  |  |  |  |  |  |  |
| Male | 85/547832 | 15.5 (12.4-19.2) | 141/977804 | 14.4 (12.1-17) | 60/706868 | 8.5 (6.5-10.9) | 80/748804 | 10.7 (8.5-13.3) | 0.004* | 0.69 (0.51-0.93) |  |
| Female | 140/549452 | 25.5 (21.4-30.1) | 134/997184 | 13.4 (11.3-15.9) | 81/698800 | 11.6 (9.2-14.4) | 81/733220 | 11 (8.8-13.7) | <0.0001* | 0.43 (0.33-0.57) |  |
| **Ethnicity** |  |  |  |  |  |  |  |  |  |  |  |
| White | 176/683076 | 25.8 (22.1-29.9) | 217/1165900 | 18.6 (16.2-21.3) | 97/800220 | 12.1 (9.8-14.8) | 104/811064 | 12.8 (10.5-15.5) | <0.0001* | 0.5 (0.39-0.63) |  |
| Black | 29/315512 | 9.2 (6.2-13.2) | 42/526272 | 8 (5.8-10.8) | 32/362668 | 8.8 (6-12.5) | 44/366448 | 12 (8.7-16.1) | 0.96 | 1.31 (0.82-2.11) |  |
| **Age groups** |  |  |  |  |  |  |  |  |  |  |  |
| <55 y | 15/932480 | 1.6 (0.9-2.7) | 30/1674408 | 1.8 (1.2-2.6) | 20/1212496 | 1.6 (1-2.5) | 15/1288640 | 1.2 (0.7-1.9) | 1 | 0.72 (0.35-1.49) |  |
| 55+ y | 210/164804 | 127.4 (110.8-145.9) | 245/300580 | 81.5 (71.6-92.4) | 121/193172 | 62.6 (52-74.8) | 146/193384 | 75.5 (63.7-88.8) | <0.0001* | 0.59 (0.48-0.73) |  |
| **Age** |  |  |  |  |  |  |  |  |  |  |  |
| <45 | 8/833532 | 1 (0.4-1.9) | 15/1459428 | 1 (0.6-1.7) | 8/1051112 | 0.8 (0.3-1.5) | 5/1104616 | 0.5 (0.1-1.1) | 0.77 | 0.47 (0.14-1.41) |  |
| 45-54 | 7/98948 | 7.1 (2.8-14.6) | 15/214980 | 7 (3.9-11.5) | 12/161384 | 7.4 (3.8-13) | 10/184024 | 5.4 (2.6-10) | 1 | 0.77 (0.3-2.12) |  |
| 55-64 | 25/67260 | 37.2 (24.1-54.9) | 19/131248 | 14.5 (8.7-22.6) | 13/90556 | 14.4 (7.6-24.5) | 27/96284 | 28 (18.5-40.8) | 1 | 0.75 (0.44-1.31) |  |
| 65-74 | 52/53156 | 97.8 (73.1-128.3) | 67/91556 | 73.2 (56.7-92.9) | 31/55956 | 55.4 (37.6-78.6) | 32/52832 | 60.6 (41.4-85.5) | 0.08 | 0.62 (0.39-0.96) |  |
| 75-84 | 80/33956 | 235.6 (186.8-293.2) | 101/57900 | 174.4 (142.1-212) | 45/34492 | 130.5 (95.2-174.6) | 46/32164 | 143 (104.7-190.8) | 0.009* | 0.61 (0.42-0.87) |  |
| 85+ | 53/10432 | 508.1 (380.6-664.5) | 58/19876 | 291.8 (221.6-377.2) | 32/12168 | 263 (179.9-371.3) | 41/12104 | 338.7 (243.1-459.5) | 0.36 | 0.67 (0.44-1) |  |
| ***Small Vessel Occlusion (SVO)*** | | | | | | | | | | | |
| **All** | 226/1097284 | 20.6 (18-23.5) | 266/1974988 | 13.5 (11.9-15.2) | 166/1405668 | 11.8 (10.1-13.7) | 127/1482024 | 8.6 (7.1-10.2) | <0.0001* | 0.42 (0.33-0.52) |  |
| **Sex** |  |  |  |  |  |  |  |  |  |  |  |
| Male | 121/547832 | 22.1 (18.3-26.4) | 150/977804 | 15.3 (13-18) | 94/706868 | 13.3 (10.7-16.3) | 73/748804 | 9.7 (7.6-12.3) | <0.0001* | 0.44 (0.33-0.59) |  |
| Female | 105/549452 | 19.1 (15.6-23.1) | 116/997184 | 11.6 (9.6-14) | 72/698800 | 10.3 (8.1-13) | 54/733220 | 7.4 (5.5-9.6) | <0.0001* | 0.39 (0.28-0.53) |  |
| **Ethnicity** |  |  |  |  |  |  |  |  |  |  |  |
| White | 141/683076 | 20.6 (17.4-24.3) | 153/1165900 | 13.1 (11.1-15.4) | 85/800220 | 10.6 (8.5-13.1) | 66/811064 | 8.1 (6.3-10.4) | <0.0001* | 0.39 (0.29-0.53) |  |
| Black | 68/315512 | 21.6 (16.7-27.3) | 85/526272 | 16.2 (12.9-20) | 65/362668 | 17.9 (13.8-22.8) | 55/366448 | 15 (11.3-19.5) | 0.61 | 0.7 (0.49-0.99) |  |
| **Age groups** |  |  |  |  |  |  |  |  |  |  |  |
| <55 y | 29/932480 | 3.1 (2.1-4.5) | 36/1674408 | 2.2 (1.5-3) | 32/1212496 | 2.6 (1.8-3.7) | 23/1288640 | 1.8 (1.1-2.7) | 0.7 | 0.57 (0.33-0.99) |  |
| 55+ y | 197/164804 | 119.5 (103.4-137.4) | 230/300580 | 76.5 (66.9-87.1) | 134/193172 | 69.4 (58.1-82.2) | 104/193384 | 53.8 (43.9-65.2) | <0.0001* | 0.45 (0.35-0.57) |  |
| **Age** |  |  |  |  |  |  |  |  |  |  |  |
| <45 | 9/833532 | 1.1 (0.5-2) | 12/1459428 | 0.8 (0.4-1.4) | 3/1051112 | 0.3 (0.1-0.8) | 6/1104616 | 0.5 (0.2-1.2) | 0.44 | 0.5 (0.17-1.4) |  |
| 45-54 | 20/98948 | 20.2 (12.3-31.2) | 24/214980 | 11.2 (7.2-16.6) | 29/161384 | 18 (12-25.8) | 17/184024 | 9.2 (5.4-14.8) | 0.66 | 0.46 (0.24-0.87) |  |
| 55-64 | 43/67260 | 63.9 (46.3-86.1) | 59/131248 | 45 (34.2-58) | 34/90556 | 37.5 (26-52.5) | 31/96284 | 32.2 (21.9-45.7) | 0.016* | 0.5 (0.31-0.8) |  |
| 65-74 | 75/53156 | 141.1 (111-176.9) | 73/91556 | 79.7 (62.5-100.3) | 36/55956 | 64.3 (45.1-89.1) | 24/52832 | 45.4 (29.1-67.6) | <0.0001* | 0.32 (0.2-0.5) |  |
| 75-84 | 54/33956 | 159 (119.5-207.5) | 69/57900 | 119.2 (92.7-150.8) | 41/34492 | 118.9 (85.3-161.3) | 35/32164 | 108.8 (75.8-151.3) | 0.57 | 0.68 (0.44-1.04) |  |
| 85+ | 25/10432 | 239.6 (155.1-353.8) | 29/19876 | 145.9 (97.7-209.5) | 23/12168 | 189 (119.8-283.6) | 14/12104 | 115.7 (63.2-194.1) | 0.48 | 0.48 (0.24-0.92) |  |
| ***Other determined aetiologies (OTH)*** | | | | | | | | | | | |
| **All** | 24/1097284 | 2.2 (1.4-3.3) | 37/1974988 | 1.9 (1.3-2.6) | 7/1405668 | 0.5 (0.2-1) | 14/1482024 | 0.9 (0.5-1.6) | 0.002* | 0.43 (0.22-0.82) |  |
| **Sex** |  |  |  |  |  |  |  |  |  |  |  |
| Male | 11/547832 | 2 (1-3.6) | 26/977804 | 2.7 (1.7-3.9) | 4/706868 | 0.6 (0.2-1.4) | 6/748804 | 0.8 (0.3-1.7) | 0.021* | 0.4 (0.14-1.05) |  |
| Female | 13/549452 | 2.4 (1.3-4) | 11/997184 | 1.1 (0.6-2) | 3/698800 | 0.4 (0.1-1.3) | 8/733220 | 1.1 (0.5-2.1) | 0.22 | 0.46 (0.18-1.09) |  |
| **Ethnicity** |  |  |  |  |  |  |  |  |  |  |  |
| White | 14/683076 | 2 (1.1-3.4) | 23/1165900 | 2 (1.3-3) | 2/800220 | 0.2 (0-0.9) | 10/811064 | 1.2 (0.6-2.3) | 0.15 | 0.6 (0.26-1.34) |  |
| Black | 10/315512 | 3.2 (1.5-5.8) | 11/526272 | 2.1 (1-3.7) | 4/362668 | 1.1 (0.3-2.8) | 1/366448 | 0.3 (0-1.5) | 0.01* | 0.09 (0-0.45) |  |
| **Age groups** |  |  |  |  |  |  |  |  |  |  |  |
| <55 y | 10/932480 | 1.1 (0.5-2) | 23/1674408 | 1.4 (0.9-2.1) | 4/1212496 | 0.3 (0.1-0.8) | 4/1288640 | 0.3 (0.1-0.8) | 0.012* | 0.29 (0.08-0.87) |  |
| 55+ y | 14/164804 | 8.5 (4.6-14.3) | 14/300580 | 4.7 (2.5-7.8) | 3/193172 | 1.6 (0.3-4.5) | 10/193384 | 5.2 (2.5-9.5) | 0.6 | 0.61 (0.26-1.36 |  |
| **Age** |  |  |  |  |  |  |  |  |  |  |  |
| <45 | 4/833532 | 0.5 (0.1-1.2) | 16/1459428 | 1.1 (0.6-1.8) | 3/1051112 | 0.3 (0.1-0.8) | 4/1104616 | 0.4 (0.1-0.9) | 0.96 | 0.75 (0.18-3.19) |  |
| 45-54 | 6/98948 | 6.1 (2.2-13.2) | 7/214980 | 3.3 (1.3-6.7) | 1/161384 | 0.6 (0-3.5) | 0/184024 | NA | 0.002* | NA |  |
| 55-64 | 7/67260 | 10.4 (4.2-21.4) | 8/131248 | 6.1 (2.6-12) | 0/90556 | NA | 5/96284 | 5.2 (1.7-12.1) | 0.48 | NA |  |
| 65-74 | 3/53156 | 5.6 (1.2-16.5) | 3/91556 | 3.3 (0.7-9.6) | 2/55956 | 3.6 (0.4-12.9) | 3/52832 | 5.7 (1.2-16.6) | 1 | 1.01 (0.19-5.44) |  |
| 75-84 | 4/33956 | 11.8 (3.2-30.2) | 3/57900 | 5.2 (1.1-15.1) | 0/34492 | NA | 1/32164 | 3.1 (0.1-17.3) | 0.43 | NA |  |
| 85+ | 0/10432 | NA | 0/19876 | NA | 1/12168 | 8.2 (0.2-45.8) | 1/12104 | 8.3 (0.2-46) | 0.97 | NA |  |
| ***Undetermined aetiologies (UND)*** | | | | | | | | | | | |
| **All** | 265/1097284 | 24.2 (21.3-27.2) | 407/1974988 | 20.6 (18.7-22.7) | 204/1405668 | 14.5 (12.6-16.6) | 196/1482024 | 13.2 (11.4-15.2) | <0.0001* | 0.55 (0.45-0.66) |  |
| **Sex** |  |  |  |  |  |  |  |  |  |  |  |
| Male | 131/547832 | 23.9 (20-28.4) | 199/977804 | 20.4 (17.6-23.4) | 99/706868 | 14 (11.4-17.1) | 106/748804 | 14.2 (11.6-17.1) | <0.0001* | 0.59 (0.46-0.76) |  |
| Female | 134/549452 | 24.4 (20.4-28.9) | 208/997184 | 20.9 (18.1-23.9) | 105/698800 | 15 (12.3-18.2) | 90/733220 | 12.3 (9.9-15.1) | <0.0001* | 0.5 (0.38-0.66) |  |
| **Ethnicity** |  |  |  |  |  |  |  |  |  |  |  |
| White | 207/683076 | 30.3 (26.3-34.7) | 291/1165900 | 25 (22.2-28) | 128/800220 | 16 (13.3-19) | 99/811064 | 12.2 (9.9-14.9) | <0.0001* | 0.4 (0.32-0.51) |  |
| Black | 33/315512 | 10.5 (7.2-14.7) | 87/526272 | 16.5 (13.2-20.4) | 55/362668 | 15.2 (11.4-19.7) | 77/366448 | 21 (16.6-26.3) | 0.015* | 2.01 (1.35-3.06) |  |
| **Age groups** |  |  |  |  |  |  |  |  |  |  |  |
| <55 y | 22/932480 | 2.4 (1.5-3.6) | 47/1674408 | 2.8 (2.1-3.7) | 46/1212496 | 3.8 (2.8-5.1) | 42/1288640 | 3.3 (2.3-4.4) | 0.76 | 1.38 (0.83-2.35) |  |
| 55+ y | 243/164804 | 147.4 (129.5-167.2) | 360/300580 | 119.8 (107.7-132.8) | 158/193172 | 81.8 (69.5-95.6) | 154/193384 | 79.6 (67.6-93.3) | <0.0001* | 0.54 (0.44-0.66) |  |
| **Age** |  |  |  |  |  |  |  |  |  |  |  |
| <45 | 6/833532 | 0.7 (0.3-1.6) | 14/1459428 | 1 (0.5-1.6) | 20/1051112 | 1.9 (1.2-2.9) | 19/1104616 | 1.7 (1-2.7) | 0.09 | 2.39 (1.01-6.56) |  |
| 45-54 | 16/98948 | 16.2 (9.2-26.3) | 33/214980 | 15.4 (10.6-21.6) | 26/161384 | 16.1 (10.5-23.6) | 23/184024 | 12.5 (7.9-18.8) | 1 | 0.77 (0.41-1.49) |  |
| 55-64 | 42/67260 | 62.4 (45-84.4) | 72/131248 | 54.9 (42.9-69.1) | 31/90556 | 34.2 (23.3-48.6) | 42/96284 | 43.6 (31.4-59) | 0.16 | 0.7 (0.45-1.07) |  |
| 65-74 | 71/53156 | 133.6 (104.3-168.5) | 100/91556 | 109.2 (88.9-132.8) | 40/55956 | 71.5 (51.1-97.3) | 40/52832 | 75.7 (54.1-103.1) | 0.002* | 0.57 (0.38-0.83) |  |
| 75-84 | 79/33956 | 232.7 (184.2-290) | 119/57900 | 205.5 (170.3-245.9) | 53/34492 | 153.7 (115.1-201) | 46/32164 | 143 (104.7-190.8) | 0.011* | 0.61 (0.42-0.88) |  |
| 85+ | 51/10432 | 488.9 (364-642.8) | 69/19876 | 347.2 (270.1-439.3) | 34/12168 | 279.4 (193.5-390.5) | 26/12104 | 214.8 (140.3-314.7) | 0.002* | 0.44 (0.27-0.7) |  |

n indicates number of cases; N, number at risk; CI, confidence interval; and IRR, incidence rate ratio. P-values were obtained from the Cochran-Armitage tests for trend after correcting for multiple comparisons.

* denotes significant trends (p<0.05).

# Standardised incidence rates

## To census population of England & Wales (2011) – EW11

**Table C: Age-adjusted trends in annual incidence per 100,000 per year (95% CI).**

|  | **2000-2003** | **2004-2007** | **2008-2011** | **2012-2015** | **P-value‡** |
| --- | --- | --- | --- | --- | --- |
| ***Overall Ischaemic Stroke (IS)*** | | | | | |
| **All** | 137.3 (127.8-147.3) | 102.5 (96.4-108.8) | 88.1 (81.1-95.7) | 78.4 (71.7-85.6) | <0.0001* |
| **Male** | 144 (129.2-160.5) | 122.7 (112.6-133.7) | 94.7 (83.6-107) | 89.3 (78.7-101.1) | <0.0001* |
| **Female** | 126.1 (114.3-138.8) | 84.7 (77.5-92.4) | 81.6 (72.6-91.3) | 67.6 (59.2-76.8) | <0.0001* |
| **White** | 127.1 (117-138) | 96.5 (89.7-103.6) | 79 (71.1-87.5) | 69.7 (61.9-78.2) | <0.0001* |
| **Black** | 144.6 (117.6-178.7) | 115.2 (100.3-132.7) | 121.4 (101.9-144.2) | 116.2 (98.5-136.9) | 0.31 |
| **<55 y** | 13.1 (10.3-16.5) | 12 (10.1-14.1) | 13.4 (11.1-16) | 8.8 (7.1-10.8) | 0.023* |
| **55+ y** | 454.3 (421.6-489) | 333.5 (312.6-355.4) | 279 (254.8-305) | 256.3 (232.8-281.5) | <0.0001* |
| ***Large Artery Atherosclerosis (LAA)*** | | | | | |
| **All** | 11.3 (8.7-14.5) | 11.9 (9.9-14.1) | 14.3 (11.5-17.5) | 6.5 (4.7-8.8) | 0.039* |
| **Male** | 12.2 (8.2-18) | 15.5 (12.1-19.8) | 14.7 (10.5-20.2) | 8.5 (5.6-12.8) | 0.15 |
| **Female** | 10.4 (7.2-14.6) | 8.7 (6.6-11.5) | 13.8 (10.3-18.2) | 4.6 (2.6-7.5) | 0.17 |
| **White** | 11.1 (8.3-14.7) | 10.8 (8.6-13.4) | 14.3 (11.1-18.2) | 6.5 (4.3-9.6) | 0.24 |
| **Black** | 8.2 (3.8-21.8) | 18 (11.9-27.3) | 19.3 (11.7-30.9) | 6.3 (2.9-13) | 0.26 |
| **<55 y** | 0.9 (0.3-2.1) | 1.7 (1-2.7) | 2.5 (1.6-3.8) | 1 (0.5-1.9) | 0.95 |
| **55+ y** | 38 (29-49.1) | 37.8 (31-45.6) | 44.2 (34.9-55.4) | 20.4 (14.2-28.5) | 0.027* |
| ***Cardio-Embolism (CE)*** | | | | | |
| **All** | 39.3 (34.3-45) | 25.9 (22.9-29.2) | 21.1 (17.7-25) | 25 (21.2-29.3) | <0.0001* |
| **Male** | 32.7 (25.8-41.3) | 30.7 (25.6-36.6) | 20.1 (15-26.5) | 27.6 (21.6-34.9) | 0.09 |
| **Female** | 41.1 (34.5-48.6) | 21.8 (18.3-25.9) | 21.5 (17-26.9) | 22.5 (17.8-28.1) | <0.0001* |
| **White** | 38 (32.5-44.2) | 27 (23.5-31) | 20.8 (16.8-25.5) | 24.7 (20.1-30.1) | <0.0001* |
| **Black** | 30.6 (18.3-51.3) | 20.6 (14.3-29.9) | 20.4 (12.9-31.8) | 28.2 (19.8-39.6) | 0.73 |
| **<55 y** | 2.1 (1.1-3.6) | 2.1 (1.4-3) | 2.1 (1.3-3.3) | 1.4 (0.8-2.4) | 0.31 |
| **55+ y** | 134.5 (116.8-154.2) | 86.7 (76.1-98.3) | 69.6 (57.7-83.4) | 85.2 (71.8-100.4) | <0.0001* |
| ***Small Vessel Occlusion (SVO)*** | | | | | |
| **All** | 37.4 (32.6-42.8) | 24.2 (21.3-27.4) | 23.6 (20-27.6) | 18 (14.9-21.6) | <0.0001* |
| **Male** | 45.1 (37.1-54.8) | 29.9 (25.2-35.5) | 30 (23.9-37.4) | 20.7 (16-26.7) | <0.0001* |
| **Female** | 31.2 (25.5-37.9) | 19 (15.7-22.8) | 18.5 (14.4-23.4) | 15.1 (11.2-19.8) | <0.0001* |
| **White** | 30.3 (25.5-35.9) | 19.4 (16.4-22.8) | 17.8 (14.2-22.1) | 14.4 (11.1-18.6) | <0.0001* |
| **Black** | 56.4 (42.4-77.5) | 35.1 (27.7-45.2) | 40 (30-53.3) | 35 (25.6-47.5) | 0.13 |
| **<55 y** | 4.8 (3.2-7.1) | 2.8 (2-4) | 3.8 (2.6-5.3) | 2.3 (1.4-3.4) | 0.07 |
| **55+ y** | 120.6 (104.3-139) | 78.7 (68.9-89.7) | 74.1 (62-88) | 58.2 (47.4-70.8) | <0.0001* |
| ***Undetermined aetiologies (UND)*** | | | | | |
| **All** | 45.7 (40.3-51.7) | 38.1 (34.4-42.1) | 28.5 (24.6-32.9) | 27.2 (23.4-31.6) | <0.0001* |
| **Male** | 50.5 (41.8-60.9) | 42.8 (36.9-49.7) | 29.1 (23.2-36.2) | 31.1 (25.1-38.4) | <0.0001* |
| **Female** | 40 (33.5-47.4) | 34 (29.5-39) | 27.3 (22.2-33.2) | 23.5 (18.7-29.1) | <0.0001* |
| **White** | 44.8 (38.9-51.5) | 36.9 (32.8-41.4) | 25.8 (21.4-30.8) | 22.2 (17.9-27.3) | <0.0001* |
| **Black** | 43.5 (27-68.8) | 38.3 (29.9-49.4) | 39.7 (28.4-54.7) | 45.9 (35-59.8) | 0.52 |
| **<55 y** | 3.7 (2.3-5.8) | 3.8 (2.7-5.1) | 4.7 (3.4-6.3) | 3.8 (2.7-5.2) | 0.79 |
| **55+ y** | 152.8 (134.1-173.6) | 125.8 (113.1-139.6) | 89.3 (75.8-104.5) | 87.1 (73.8-102.3) | <0.0001* |

‡P-values were obtained from the Cochran-Armitage tests for trend. * denotes significant trends (p<0.05).

## To European Standard Population (2013) – ESP13

**Table D: Age-adjusted trends in annual incidences per 100,000 per year (95% CI).**

|  | **2000-2003** | **2004-2007** | **2008-2011** | **2012-2015** | **P-value‡** |
| --- | --- | --- | --- | --- | --- |
| ***Overall Ischaemic Stroke (IS)*** | | | | | |
| **All** | 155.9 (145.1-167.2) | 116.4 (109.5-123.6) | 99.5 (91.5-108.1) | 88.7 (81-96.9) | <0.0001* |
| **Male** | 163.8 (147-182.5) | 138.5 (127.1-150.9) | 107 (94.4-121) | 101 (88.9-114.5) | <0.0001* |
| **Female** | 143.2 (129.8-157.6) | 96.9 (88.7-105.8) | 92.2 (82-103.3) | 76.5 (66.9-87) | <0.0001* |
| **White** | 144.5 (133-156.8) | 109 (101.4-117.1) | 89.1 (80.1-98.8) | 79.1 (70.2-89) | <0.0001* |
| **Black** | 163.5 (133.1-201.9) | 133.4 (116.1-153.7) | 137.6 (115.6-163.5) | 130 (110.2-153) | 0.22 |
| **<55 y** | 14.4 (11.3-18.1) | 13 (10.9-15.3) | 14.4 (11.9-17.3) | 9.4 (7.5-11.5) | 0.014* |
| **55+ y** | 456.6 (423.8-491.3) | 336.2 (315.2-358.3) | 280.5 (256.1-306.7) | 257.3 (233.7-282.8) | <0.0001* |
| ***Large Artery Atherosclerosis (LAA)*** | | | | | |
| **All** | 12.9 (9.9-16.5) | 13.7 (11.4-16.3) | 16.2 (13-19.8) | 7.3 (5.3-10) | 0.036* |
| **Male** | 14.1 (9.5-20.7) | 17.7 (13.9-22.6) | 16.7 (11.9-22.9) | 9.7 (6.2-14.6) | 0.15 |
| **Female** | 11.7 (8-16.4) | 10.2 (7.7-13.4) | 15.6 (11.6-20.6) | 5.2 (3-8.6) | 0.17 |
| **White** | 12.7 (9.4-16.7) | 12.3 (9.8-15.3) | 16.2 (12.5-20.7) | 7.5 (4.9-11) | 0.24 |
| **Black** | 9.2 (4.2-24.5) | 21.1 (14-31.7) | 22 (13.3-35.2) | 6.9 (3.1-14.5) | 0.23 |
| **<55 y** | 1 (0.3-2.4) | 1.9 (1.2-3) | 2.7 (1.7-4.1) | 1.1 (0.5-2) | 0.91 |
| **55+ y** | 38.2 (29.2-49.3) | 38.7 (31.8-46.7) | 44.7 (35.3-56) | 20.7 (14.4-28.9) | 0.028* |
| ***Cardio-Embolism (CE)*** | | | | | |
| **All** | 44.5 (38.8-50.8) | 29.6 (26.1-33.4) | 24 (20.1-28.5) | 28.4 (24-33.3) | <0.0001* |
| **Male** | 36.9 (29.1-46.6) | 34.9 (29.1-41.7) | 23 (17.2-30.3) | 31.4 (24.6-39.7) | 0.1 |
| **Female** | 46.6 (39.1-55.1) | 25 (20.9-29.8) | 24.4 (19.3-30.6) | 25.4 (20-31.8) | <0.0001* |
| **White** | 43.2 (37-50.2) | 30.9 (26.8-35.3) | 23.8 (19.2-29.2) | 28.2 (22.9-34.4) | <0.0001* |
| **Black** | 34.3 (20.5-57.3) | 23.5 (16.4-34) | 23.4 (14.8-36.2) | 31.8 (22.4-44.6) | 0.73 |
| **<55 y** | 2.2 (1.2-3.9) | 2.2 (1.4-3.3) | 2.2 (1.3-3.5) | 1.6 (0.9-2.6) | 0.4 |
| **55+ y** | 134.2 (116.6-153.9) | 87.7 (77-99.5) | 70.3 (58.3-84.2) | 85.3 (71.9-100.7) | <0.0001* |
| ***Small Vessel Occlusion (SVO)*** | | | | | |
| **All** | 42.7 (37.2-48.8) | 27.6 (24.3-31.2) | 26.6 (22.6-31.2) | 20.3 (16.7-24.4) | <0.0001* |
| **Male** | 51.7 (42.6-62.8) | 34 (28.6-40.4) | 33.8 (26.9-42.1) | 23.5 (18.1-30.4) | <0.0001* |
| **Female** | 35.4 (28.9-43) | 21.7 (17.9-26.1) | 20.9 (16.3-26.6) | 16.8 (12.5-22.2) | <0.0001* |
| **White** | 34.5 (29-40.7) | 22 (18.6-25.8) | 20 (15.9-24.8) | 16.3 (12.4-21.1) | <0.0001* |
| **Black** | 64.6 (48.5-88.7) | 40.8 (32.1-52.5) | 45.3 (34-60.4) | 39.1 (28.6-53) | 0.09 |
| **<55 y** | 5.3 (3.5-7.8) | 3.1 (2.2-4.4) | 4.2 (2.8-5.9) | 2.5 (1.6-3.8) | 0.06 |
| **55+ y** | 122.2 (105.6-140.7) | 79.5 (69.5-90.6) | 74.3 (62.1-88.2) | 58.1 (47.3-70.7) | <0.0001* |
| ***Undetermined aetiologies (UND)*** | | | | | |
| **All** | 52 (45.9-58.8) | 43 (38.9-47.5) | 32 (27.6-37) | 30.9 (26.5-35.9) | <0.0001* |
| **Male** | 57.3 (47.4-69) | 47.8 (41.1-55.4) | 32.6 (25.9-40.7) | 35.1 (28.1-43.4) | <0.0001* |
| **Female** | 45.7 (38.3-54.2) | 38.8 (33.6-44.5) | 30.7 (25-37.5) | 26.9 (21.4-33.5) | <0.0001* |
| **White** | 50.9 (44.2-58.5) | 41.4 (36.8-46.5) | 28.8 (23.9-34.5) | 25.3 (20.3-31.2) | <0.0001* |
| **Black** | 49.4 (30.8-77.6) | 44.5 (34.7-57.3) | 44.8 (32.1-61.7) | 51.2 (39-66.6) | 0.64 |
| **<55 y** | 4.2 (2.5-6.4) | 4.1 (3-5.5) | 5 (3.6-6.8) | 4 (2.8-5.5) | 0.97 |
| **55+ y** | 153.7 (134.9-174.6) | 125.8 (113.1-139.6) | 89.4 (75.9-104.6) | 88.1 (74.5-103.5) | <0.0001* |

‡P-values were obtained from the Cochran-Armitage tests for trend. * denotes significant trends (p<0.05).

**Cont.’ table D.**

**
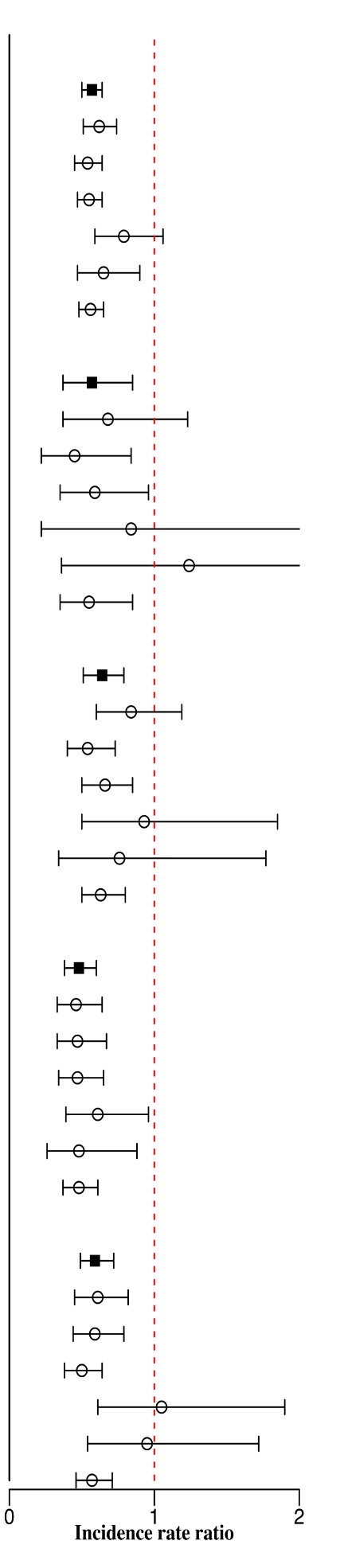
**

|  | **2000-2003** | **2012-2015** | **IRR (95% CI)** |
| --- | --- | --- | --- |
| ***Overall Ischaemic Stroke (IS)*** | | | |
| **All** | 155.9 (145.1-167.2) | 88.7 (81-96.9) | 0.57 (0.5-0.64) |
| **Male** | 163.8 (147-182.5) | 101 (88.9-114.5) | 0.62 (0.51-0.74) |
| **Female** | 143.2 (129.8-157.6) | 76.5 (66.9-87) | 0.54 (0.45-0.64) |
| **White** | 144.5 (133-156.8) | 79.1 (70.2-89) | 0.55 (0.47-0.64) |
| **Black** | 163.5 (133.1-201.9) | 130 (110.2-153) | 0.79 (0.59-1.06) |
| **<55 y** | 14.4 (11.3-18.1) | 9.4 (7.5-11.5) | 0.65 (0.47-0.9) |
| **55+ y** | 456.6 (423.8-491.3) | 257.3 (233.7-282.8) | 0.56 (0.48-0.65) |
| ***Large Artery Atherosclerosis (LAA)*** | | | |
| **All** | 12.9 (9.9-16.5) | 7.3 (5.3-10) | 0.57 (0.37-0.85) |
| **Male** | 14.1 (9.5-20.7) | 9.7 (6.2-14.6) | 0.68 (0.37-1.23) |
| **Female** | 11.7 (8-16.4) | 5.2 (3-8.6) | 0.45 (0.22-0.84) |
| **White** | 12.7 (9.4-16.7) | 7.5 (4.9-11) | 0.59 (0.35-0.96) |
| **Black** | 9.2 (4.2-24.5) | 6.9 (3.1-14.5) | 0.84 (0.22-3.98) |
| **<55 y** | 1 (0.3-2.4) | 1.1 (0.5-2) | 1.24 (0.36-5.65) |
| **55+ y** | 38.2 (29.2-49.3) | 20.7 (14.4-28.9) | 0.55 (0.35-0.85) |
| ***Cardio-Embolism (CE)*** | | | |
| **All** | 44.5 (38.8-50.8) | 28.4 (24-33.3) | 0.64 (0.51-0.79) |
| **Male** | 36.9 (29.1-46.6) | 31.4 (24.6-39.7) | 0.84 (0.6-1.19) |
| **Female** | 46.6 (39.1-55.1) | 25.4 (20-31.8) | 0.54 (0.4-0.73) |
| **White** | 43.2 (37-50.2) | 28.2 (22.9-34.4) | 0.66 (0.5-0.85) |
| **Black** | 34.3 (20.5-57.3) | 31.8 (22.4-44.6) | 0.93 (0.5-1.85) |
| **<55 y** | 2.2 (1.2-3.9) | 1.6 (0.9-2.6) | 0.76 (0.34-1.77) |
| **55+ y** | 134.2 (116.6-153.9) | 85.3 (71.9-100.7) | 0.63 (0.5-0.8) |
| ***Small Vessel Occlusion (SVO)*** | | | |
| **All** | 42.7 (37.2-48.8) | 20.3 (16.7-24.4) | 0.48 (0.38-0.6) |
| **Male** | 51.7 (42.6-62.8) | 23.5 (18.1-30.4) | 0.46 (0.33-0.64) |
| **Female** | 35.4 (28.9-43) | 16.8 (12.5-22.2) | 0.47 (0.33-0.67) |
| **White** | 34.5 (29-40.7) | 16.3 (12.4-21.1) | 0.47 (0.34-0.65) |
| **Black** | 64.6 (48.5-88.7) | 39.1 (28.6-53) | 0.61 (0.39-0.96) |
| **<55 y** | 5.3 (3.5-7.8) | 2.5 (1.6-3.8) | 0.48 (0.26-0.88) |
| **55+ y** | 122.2 (105.6-140.7) | 58.1 (47.3-70.7) | 0.48 (0.37-0.61) |
| ***Undetermined aetiologies (UND)*** | | | |
| **All** | 52 (45.9-58.8) | 30.9 (26.5-35.9) | 0.59 (0.49-0.72) |
| **Male** | 57.3 (47.4-69) | 35.1 (28.1-43.4) | 0.61 (0.45-0.82) |
| **Female** | 45.7 (38.3-54.2) | 26.9 (21.4-33.5) | 0.59 (0.44-0.79) |
| **White** | 50.9 (44.2-58.5) | 25.3 (20.3-31.2) | 0.5 (0.38-0.64) |
| **Black** | 49.4 (30.8-77.6) | 51.2 (39-66.6) | 1.05 (0.61-1.9) |
| **<55 y** | 4.2 (2.5-6.4) | 4 (2.8-5.5) | 0.95 (0.54-1.72) |
| **55+ y** | 153.7 (134.9-174.6) | 88.1 (74.5-103.5) | 0.57 (0.46-0.71) |

‡P-values were obtained from the Cochran-Armitage tests for trend. * denotes significant trends (p<0.05). IRR indicates incidence rate ratio (2012-15 vs 2000-03); and CI, confidence interval.

**Fig A: Trends in age-adjusted incidences, to ESP13, by demographic subgroups.**


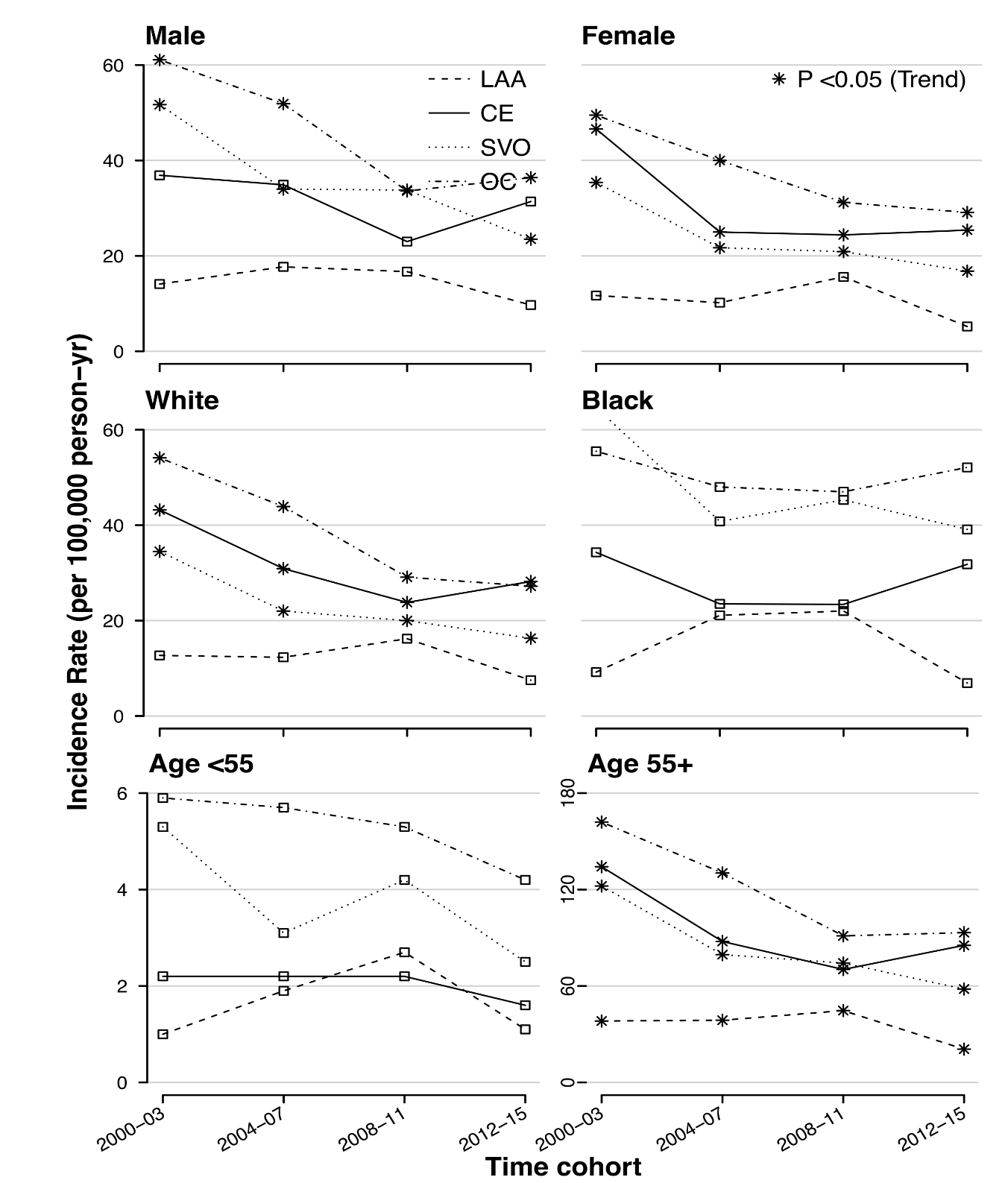


LAA indicates large artery atherosclerosis; CE, cardio-embolism; SVO, small vessel occlusion; and OC, other causes. P-values were obtained from the Cochran-Armitage tests for trend. * denotes significant trends (p<0.05).

**Fig B: Trends in age-adjusted incidences, to ESP13, by TOAST aetiological subtypes.**


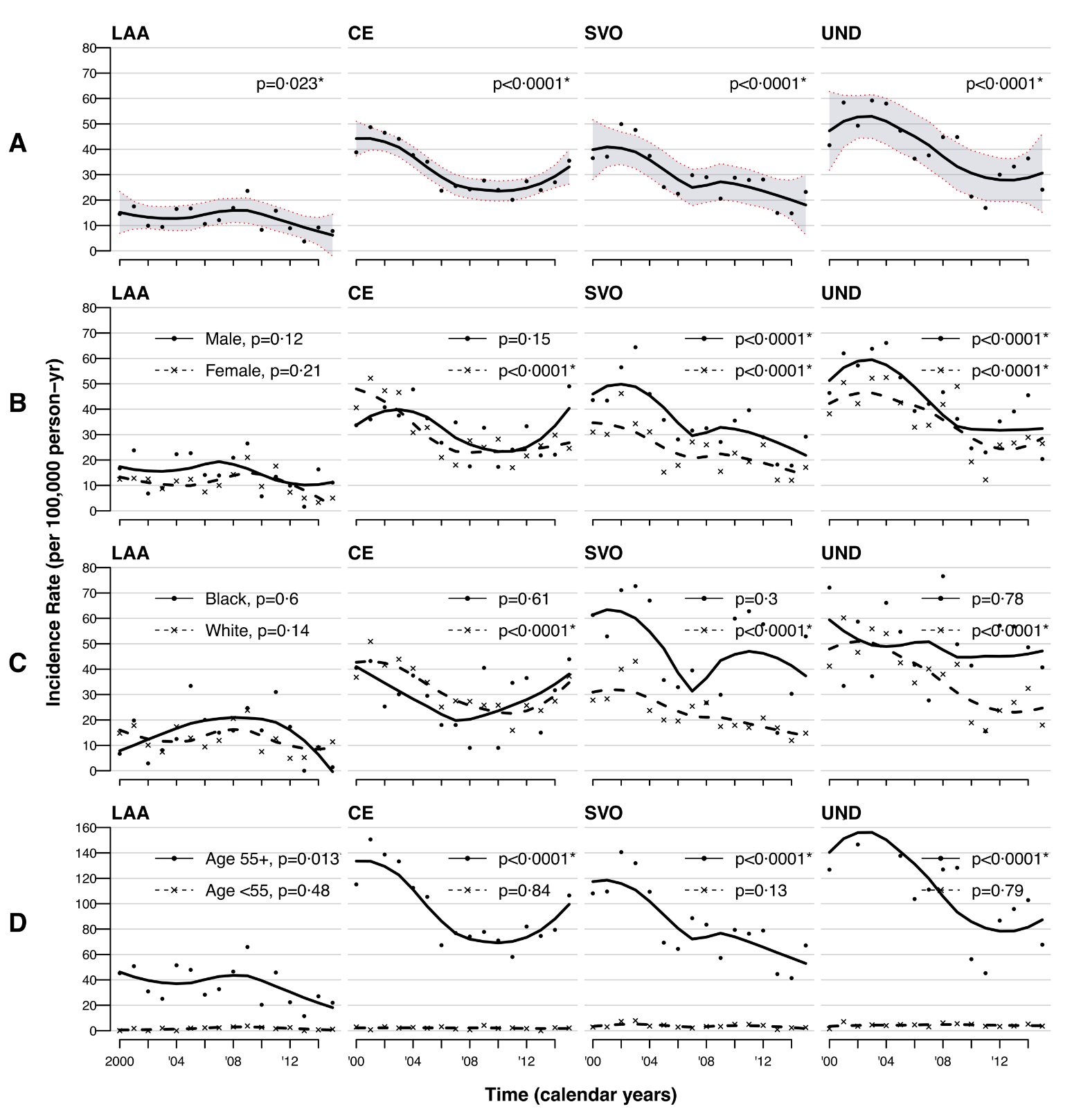
Data are the observed values with regression fitted lines (loess). LAA indicates large artery atherosclerosis; CE, cardio-embolism; SVO, small vessel occlusion; and UND, undetermined aetiologies. P-values were obtained from the Cochran-Armitage tests for trend. * denotes significant trends (p<0.05).

# Trends in risk factors in male patients

**Table E: Premorbid risk factors and medication use in males with incident ischaemic stroke.**

|  | **2000-2003 (n=380)** | **2004-2007 (n=593)** | **2008-2011 (n=304)** | **2012-2015 (n=295)** | **P-value (trend)†** | **Adjusted model‡** |
| --- | --- | --- | --- | --- | --- | --- |
|  |  |  |  |  |  | **OR (95% CI)** |
| ***Premorbid risk factors*** | | | | | | |
| **Current drinker** | 241 (67.5) | 384 (72.6) | 147 (54.6) | 157 (57.3) | <0.0001* | 0.69 (0.49-0.97) * |
| **Smoker** | 140 (39.1) | 201 (36.5) | 104 (36.7) | 85 (30.4) | 0.035* | 0.65 (0.46-0.92) * |
| **Hypertension** | 230 (62.2) | 397 (67.3) | 188 (63.3) | 195 (67.5) | 0.35 | 1.23 (0.88-1.73) |
| **Diabetes mellitus** | 76 (20.8) | 142 (24.2) | 71 (24.0) | 73 (25.1) | 0.23 | 1.15 (0.79-1.68) |
| **Hypercholesterolaemia** | 55 (15.3) | 164 (27.8) | 96 (32.9) | 133 (46.3) | <0.0001* | 4.87 (3.36-7.07) * |
| **Atrial fibrillation** | 44 (11.9) | 81 (13.8) | 39 (13.3) | 56 (19.4) | 0.013* | 2.21 (1.42-3.44) * |
| **Myocardial infarction** | 52 (14.0) | 69 (11.7) | 22 ( 7.7) | 32 (11.4) | 0.11 | 0.87 (0.54-1.41) |
| **TIA** | 42 (11.3) | 63 (10.7) | 23 ( 7.9) | 26 ( 9.1) | 0.18 | 0.64 (0.34-1.21) |
| ***Premorbid medication*** | | | | | | |
| **Antihypertensive** | 168 (45.9) | 303 (51.5) | 93 (32.2) | 91 (31.3) | <0.0001* | 0.48 (0.34-0.67) * |
| **Anti-diabetic** | 76 (20.3) | 144 (24.3) | 61 (20.5) | 59 (20.3) | 0.67 | 0.87 (0.59-1.29) |
| **Antiplatelet** | 123 (44.7) | 231 (47.7) | 99 (34.7) | 81 (27.9) | <0.0001* | 0.6 (0.42-0.87) * |
| **Anticoagulant** | 15 ( 5.5) | 24 ( 4.1) | 11 ( 3.8) | 20 ( 6.9) | 0.4 | 1.52 (0.73-3.15) |
| **Cholesterol-lowering** | 50 (16.2) | 170 (28.9) | 113 (39.2) | 105 (36.1) | <0.0001* | 3.5 (2.37-5.15) * |

OR indicates odds ratio; CI, confidence interval; and TIA, transient ischaemic attack. † Cochran-Armitage tests for trend. ‡ 2012-15 with reference to 2000-03 after adjusting for age and ethnicity and allowing for an interaction between time and ethnicity as appropriate.

**Fig C: Prior risk factors and medication use over time in male patients with first-ever ischaemic stroke.**

**

**

P-values were obtained for the unadjusted rates by the Cochran-Armitage tests for trend. Adjusted rates were computed by multiplying the adjusted coefficients in each time period by the baseline rate in the 2000-2003 cohort. Adjustments were made for age and ethnicity with an interaction term between time in years and ethnicity as appropriate.

# Trends in risk factors in female patients

**Table F: Premorbid risk factors and medication use in females with incident ischaemic stroke.**

|  | **2000-2003 (n=426)** | **2004-2007 (n=524)** | **2008-2011 (n=316)** | **2012-2015 (n=250)** | **P-value (trend)†** | **Adjusted model‡** |
| --- | --- | --- | --- | --- | --- | --- |
|  |  |  |  |  |  | **OR (95% CI)** |
| ***Premorbid risk factors*** | | | | | | |
| **Current drinker** | 154 (38.6) | 201 (42.9) | 100 (36.6) | 59 (27.4) | 0.005* | 0.82 (0.52-1.29) |
| **Smoker** | 83 (21.4) | 101 (20.9) | 62 (21.8) | 32 (14.5) | 0.1 | 0.74 (0.43-1.28) |
| **Hypertension** | 253 (59.8) | 360 (69.1) | 218 (70.6) | 184 (73.9) | <0.0001* | 1.79 (1.15-2.79) * |
| **Diabetes mellitus** | 78 (18.5) | 107 (20.6) | 76 (24.1) | 64 (26.1) | 0.01* | 1.27 (0.77-2.12) |
| **Hypercholesterolaemia** | 60 (14.5) | 134 (25.7) | 103 (33.3) | 122 (49.4) | <0.0001* | 6.57 (4.14-10.43) * |
| **Atrial fibrillation** | 100 (23.6) | 89 (17.1) | 55 (17.8) | 72 (29.3) | 0.23 | 1.33 (0.86-2.07) |
| **Myocardial infarction** | 42 (10.0) | 41 ( 7.9) | 16 ( 5.2) | 24 (10.1) | 0.46 | 1.05 (0.56-1.96) |
| **TIA** | 55 (13.0) | 54 (10.4) | 33 (10.6) | 30 (12.1) | 0.64 | 1.1 (0.63-1.91) |
| ***Premorbid medication*** | | | | | | |
| **Antihypertensive** | 203 (49.0) | 287 (55.5) | 110 (36.1) | 101 (41.7) | 0.0006* | 0.69 (0.46-1.05) |
| **Anti-diabetic** | 78 (18.3) | 108 (20.7) | 60 (19.0) | 53 (21.6) | 0.43 | 0.89 (0.51-1.54) |
| **Antiplatelet** | 133 (40.4) | 195 (42.3) | 123 (40.2) | 81 (33.5) | 0.1 | 0.87 (0.56-1.35) |
| **Anticoagulant** | 15 ( 4.6) | 19 ( 3.7) | 15 ( 4.9) | 16 ( 6.6) | 0.2 | 1.02 (0.42-2.48) |
| **Cholesterol-lowering** | 41 (11.7) | 128 (24.8) | 115 (37.6) | 95 (39.1) | <0.0001* | 5.75 (3.46-9.57) * |

OR indicates odds ratio; CI, confidence interval; and TIA, transient ischaemic attack. † Cochran-Armitage tests for trend. ‡ 2012-15 with reference to 2000-03 after adjusting for age and ethnicity and allowing for an interaction between time and ethnicity as appropriate.

**Fig D: Prior risk factors and medication use over time in female patients with first-ever ischaemic stroke.**


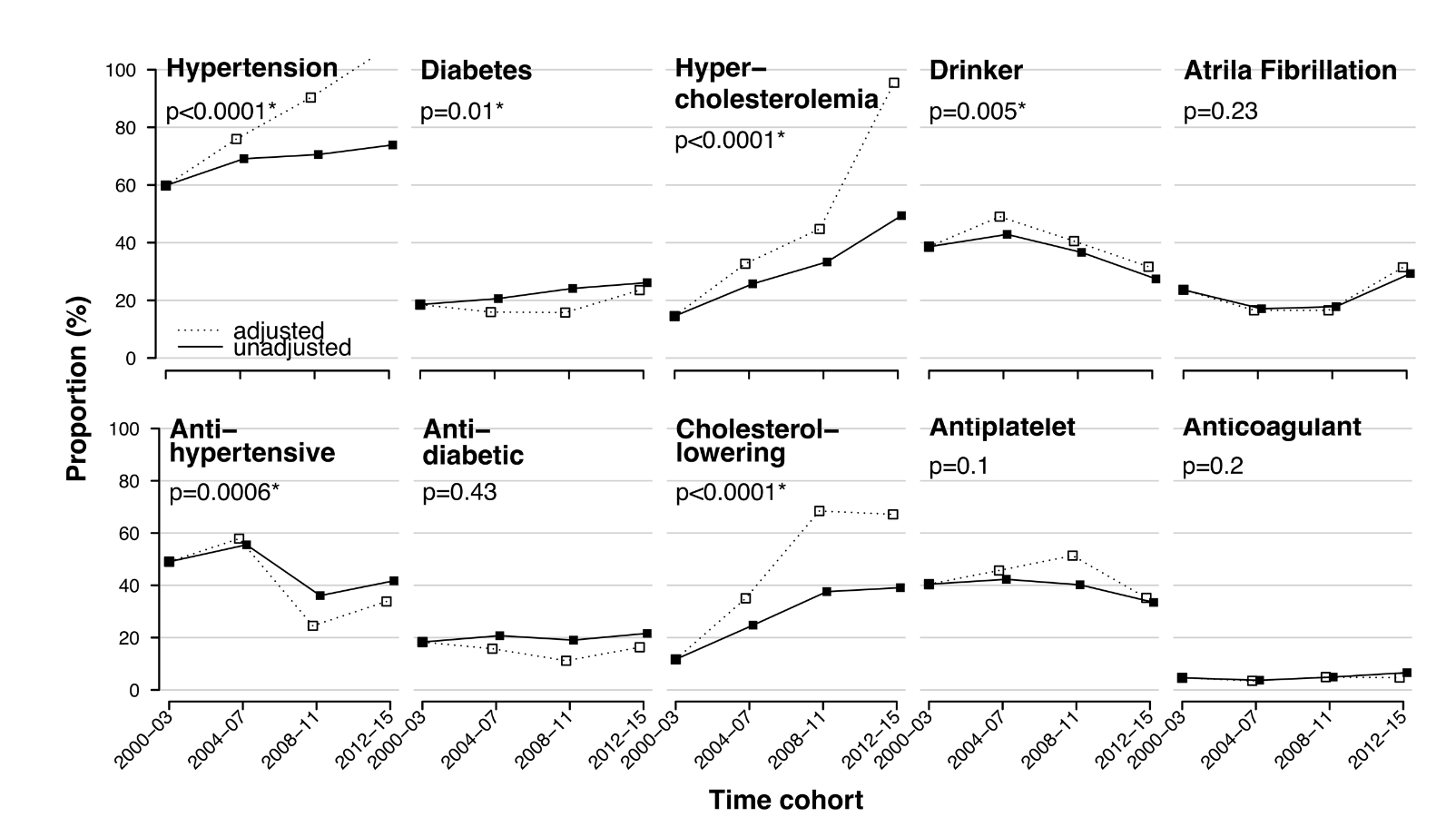


P-values were obtained for the unadjusted rates by the Cochran-Armitage tests for trend. Adjusted rates were computed by multiplying the adjusted coefficients in each time period by the baseline rate in the 2000-2003 cohort. Adjustments were made for age and ethnicity with an interaction term between time in years and ethnicity as appropriate.

# Trends in risk factors in white patients

**Table G: Premorbid risk factors and medication use in white patients with incident ischaemic stroke.**

|  | **2000-2003 (n=589)** | **2004-2007 (n=769)** | **2008-2011 (n=381)** | **2012-2015 (n=308)** | **P-value (trend)†** | **Adjusted model‡** |
| --- | --- | --- | --- | --- | --- | --- |
|  |  |  |  |  |  | **OR (95% CI)** |
| ***Premorbid risk factors*** | | | | | | |
| **Current drinker** | 297 (53.6) | 425 (62.6) | 172 (51.7) | 143 (52.8) | 0.36 | 0.78 (0.57-1.08) |
| **Smoker** | 168 (30.8) | 235 (33.2) | 115 (33.3) | 75 (26.9) | 0.44 | 0.63 (0.45-0.88) * |
| **Hypertension** | 347 (59.8) | 493 (64.4) | 232 (62.4) | 207 (68.3) | 0.032* | 1.6 (1.18-2.16) * |
| **Diabetes mellitus** | 94 (16.4) | 128 (16.8) | 55 (14.5) | 59 (19.4) | 0.56 | 1.25 (0.87-1.79) |
| **Hypercholesterolaemia** | 84 (14.9) | 212 (27.7) | 119 (32.1) | 148 (49.2) | <0.0001* | 5.44 (3.93-7.53) * |
| **Atrial fibrillation** | 127 (21.9) | 147 (19.2) | 65 (17.3) | 87 (29.0) | 0.12 | 1.7 (1.22-2.35) * |
| **Myocardial infarction** | 76 (13.1) | 87 (11.4) | 23 ( 6.2) | 42 (14.2) | 0.39 | 1.06 (0.71-1.61) |
| **TIA** | 84 (14.5) | 91 (11.9) | 34 ( 9.1) | 37 (12.3) | 0.1 | 0.85 (0.56-1.3) |
| ***Premorbid medication*** | | | | | | |
| **Antihypertensive** | 265 (46.6) | 381 (50.2) | 104 (28.3) | 90 (29.8) | <0.0001* | 0.52 (0.38-0.7) * |
| **Anti-diabetic** | 94 (16.1) | 131 (17.1) | 43 (11.3) | 45 (14.8) | 0.16 | 0.89 (0.6-1.31) |
| **Antiplatelet** | 198 (44.1) | 315 (47.5) | 152 (41.5) | 98 (32.6) | 0.0007* | 0.71 (0.52-0.97) * |
| **Anticoagulant** | 28 ( 6.3) | 39 ( 5.1) | 19 ( 5.2) | 22 ( 7.3) | 0.63 | 1.19 (0.66-2.15) |
| **Cholesterol-lowering** | 68 (14.2) | 211 (27.8) | 144 (39.2) | 115 (38.0) | <0.0001* | 4.21 (2.98-5.95) * |

OR indicates odds ratio; CI, confidence interval; and TIA, transient ischaemic attack. † Cochran-Armitage tests for trend. ‡ 2012-15 with reference to 2000-03 after adjusting for age and sex.

**Fig E: Prior risk factors and medication use over time in white patients with first-ever ischaemic stroke.**


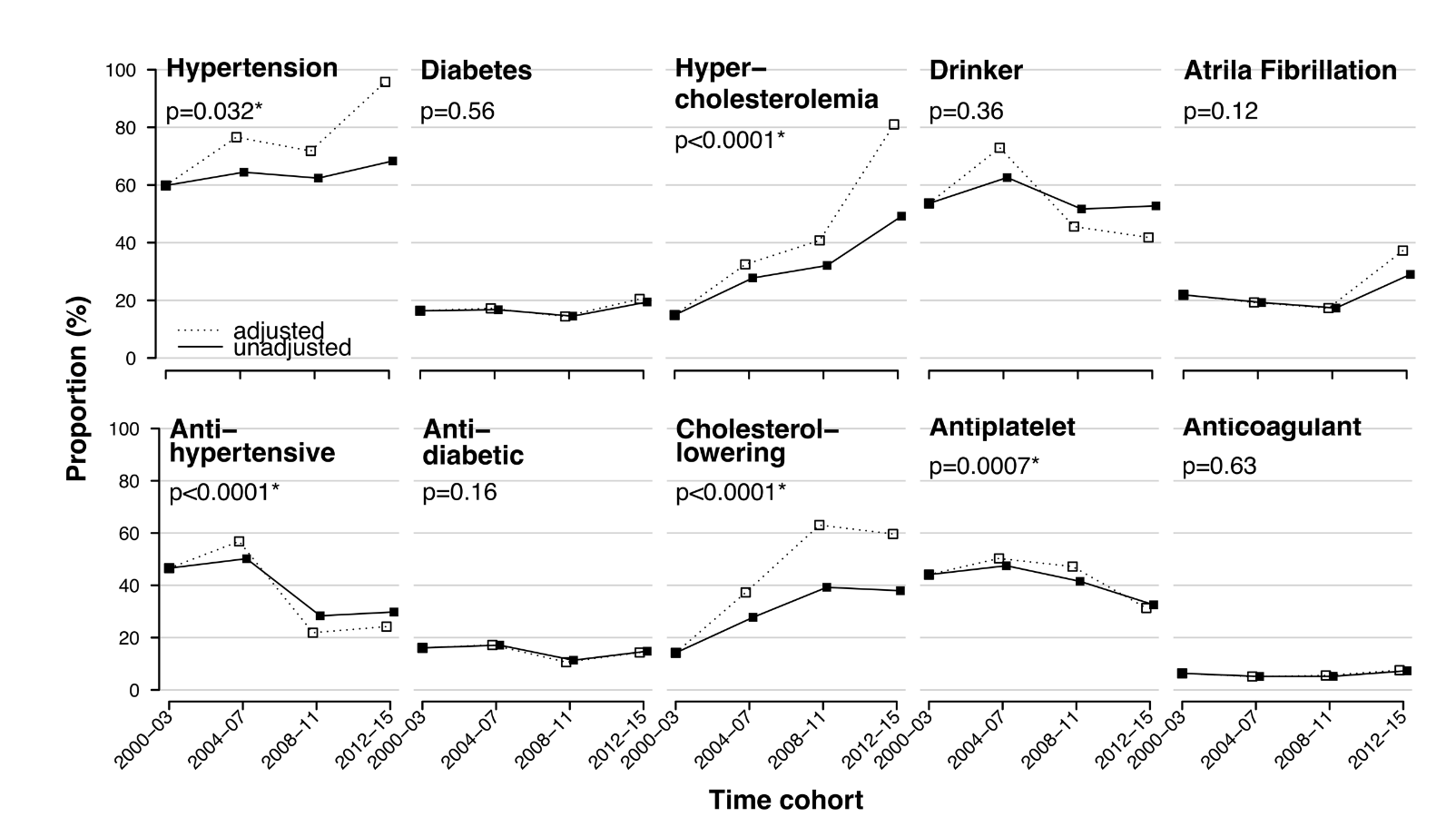


P-values were obtained for the unadjusted rates by the Cochran-Armitage tests for trend. Adjusted rates were computed by multiplying the adjusted coefficients in each time period by the baseline rate in the 2000-2003 cohort. Adjustments were made for age and sex.

# Trends in risk factors in black patients

**Table H: Premorbid risk factors and medication use in black patients with incident ischaemic stroke.**

|  | **2000-2003 (n=150)** | **2004-2007 (n=262)** | **2008-2011 (n=184)** | **2012-2015 (n=190)** | **P-value (trend)†** | **Adjusted model‡** |
| --- | --- | --- | --- | --- | --- | --- |
|  |  |  |  |  |  | **OR (95% CI)** |
| ***Premorbid risk factors*** | | | | | | |
| **Current drinker** | 72 (49.3) | 136 (56.4) | 63 (37.5) | 66 (37.1) | 0.0004* | 0.56 (0.36-0.89) * |
| **Smoker** | 34 (23.4) | 48 (19.4) | 36 (21.1) | 30 (16.8) | 0.22 | 0.62 (0.35-1.08) |
| **Hypertension** | 100 (67.1) | 208 (80.0) | 137 (75.7) | 141 (75.0) | 0.36 | 1.49 (0.91-2.45) |
| **Diabetes mellitus** | 46 (31.5) | 92 (35.9) | 71 (39.4) | 61 (32.8) | 0.76 | 1.02 (0.63-1.63) |
| **Hypercholesterolaemia** | 22 (15.4) | 62 (23.8) | 63 (35.2) | 83 (44.6) | <0.0001* | 4.64 (2.69-8.01) * |
| **Atrial fibrillation** | 10 ( 6.8) | 17 ( 6.5) | 21 (12.0) | 34 (18.1) | <0.0001* | 3.04 (1.43-6.44) * |
| **Myocardial infarction** | 10 ( 6.8) | 14 ( 5.4) | 11 ( 6.3) | 10 ( 5.6) | 0.79 | 0.73 (0.29-1.82) |
| **TIA** | 6 ( 4.0) | 21 ( 8.0) | 16 ( 9.0) | 15 ( 8.0) | 0.2 | 2.02 (0.76-5.37) |
| ***Premorbid medication*** | | | | | | |
| **Antihypertensive** | 76 (52.1) | 171 (65.5) | 85 (47.8) | 83 (44.9) | 0.004* | 0.72 (0.46-1.13) |
| **Anti-diabetic** | 46 (31.1) | 92 (35.2) | 60 (33.3) | 51 (27.4) | 0.31 | 0.78 (0.48-1.26) |
| **Antiplatelet** | 36 (36.4) | 84 (39.1) | 54 (30.5) | 49 (26.5) | 0.012* | 0.67 (0.39-1.16) |
| **Anticoagulant** | 1 ( 1.0) | 3 ( 1.1) | 5 ( 2.8) | 11 ( 5.9) | 0.002* | 3.96 (0.71-22.19) |
| **Cholesterol-lowering** | 15 (12.2) | 63 (24.1) | 64 (36.0) | 66 (35.7) | <0.0001* | 4.54 (2.44-8.46) * |

OR indicates odds ratio; CI, confidence interval; and TIA, transient ischaemic attack. † Cochran-Armitage tests for trend. ‡ 2012-15 with reference to 2000-03 after adjusting for age and sex.

**Fig F: Prior risk factors and medication use over time in black patients with first-ever ischaemic stroke.**


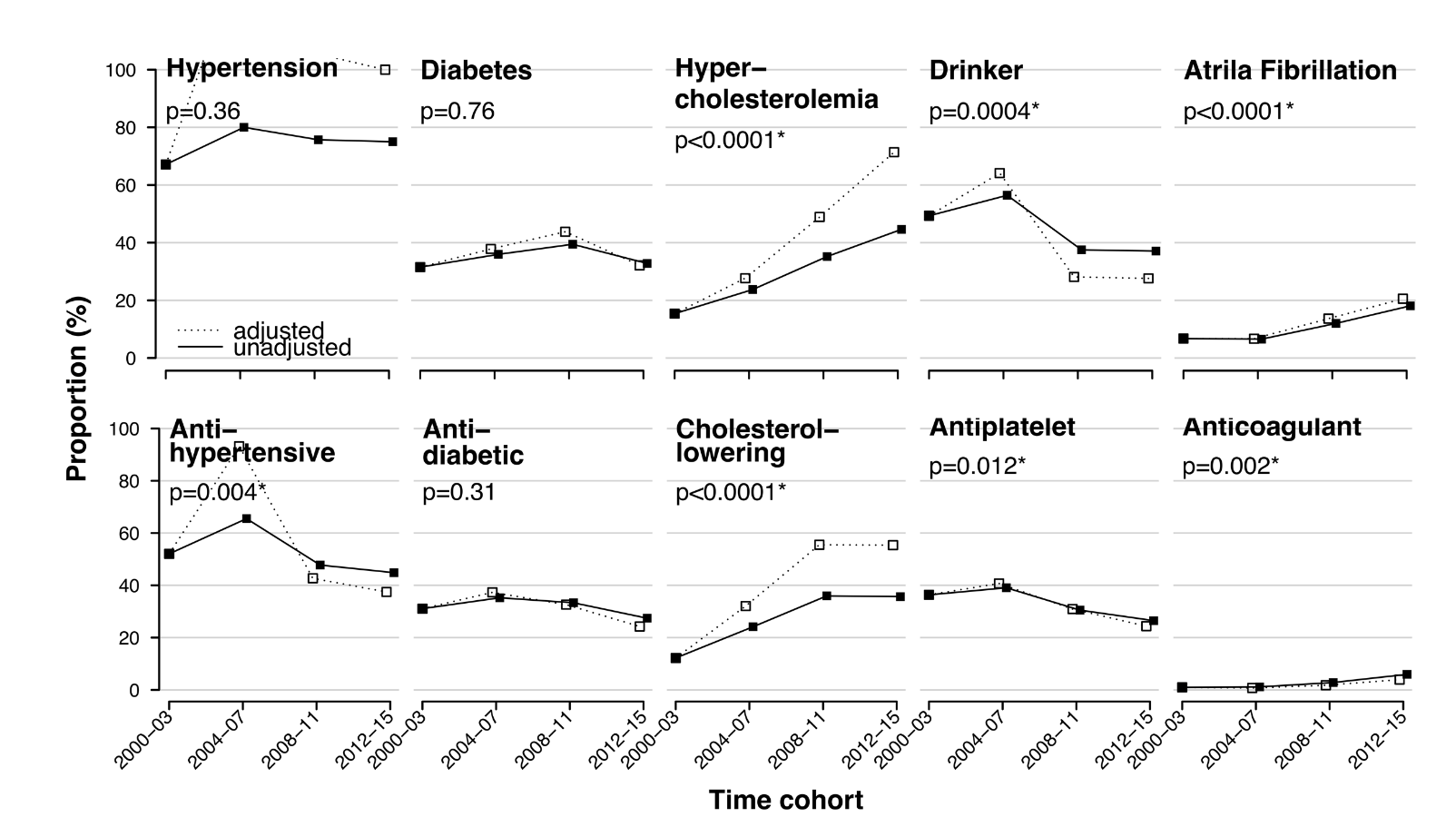


P-values were obtained for the unadjusted rates by the Cochran-Armitage tests for trend. Adjusted rates were computed by multiplying the adjusted coefficients in each time period by the baseline rate in the 2000-2003 cohort. Adjustments were made for age and sex.

# Trends in risk factors in patients <55 years

**Table I: Premorbid risk factors and medication use in patients with an incident ischaemic stroke at age <55y.**

|  | **2000-2003 (n=81)** | **2004-2007 (n=157)** | **2008-2011 (n=126)** | **2012-2015 (n=95)** | **P-value (trend)†** | **Adjusted model‡** |
| --- | --- | --- | --- | --- | --- | --- |
|  |  |  |  |  |  | **OR (95% CI)** |
| ***Premorbid risk factors*** | | | | | | |
| **Current drinker** | 45 (57.7) | 94 (67.6) | 60 (52.2) | 42 (46.7) | 0.017* | 0.64 (0.34-1.22) |
| **Smoker** | 33 (42.9) | 62 (43.4) | 43 (35.5) | 27 (29.7) | 0.029* | 0.57 (0.29-1.12) |
| **Hypertension** | 28 (35.4) | 67 (42.9) | 60 (47.6) | 47 (49.5) | 0.048* | 1.98 (1.02-3.84) * |
| **Diabetes mellitus** | 9 (12.2) | 18 (11.7) | 22 (17.5) | 18 (18.9) | 0.09 | 1.64 (0.69-3.91) |
| **Hypercholesterolaemia** | 8 (10.8) | 27 (17.3) | 22 (17.7) | 34 (35.8) | <0.0001* | 6.1 (2.5-14.89) * |
| **Atrial fibrillation** | 5 ( 6.4) | 6 ( 3.8) | 6 ( 4.8) | 11 (11.7) | 0.1 | 2.13 (0.7-6.53) |
| **Myocardial infarction** | 4 ( 5.1) | 4 ( 2.6) | 2 ( 1.7) | 8 ( 9.1) | 0.22 | 1.99 (0.56-7.13) |
| **TIA** | 3 ( 3.9) | 7 ( 4.5) | 13 (10.4) | 4 ( 4.3) | 0.43 | 1.3 (0.27-6.23) |
| ***Premorbid medication*** | | | | | | |
| **Antihypertensive** | 18 (23.1) | 42 (26.9) | 28 (23.1) | 18 (19.4) | 0.37 | 0.17 (0.03-0.85) * |
| **Anti-diabetic** | 9 (11.5) | 18 (11.5) | 21 (16.7) | 12 (12.6) | 0.52 | 0.98 (0.39-2.47) |
| **Antiplatelet** | 7 (14.3) | 20 (20.2) | 14 (11.6) | 13 (14.0) | 0.42 | 1.1 (0.38-3.17) |
| **Anticoagulant** | 4 ( 7.7) | 3 ( 1.9) | 2 ( 1.7) | 6 ( 6.5) | 0.77 | 1.01 (0.27-3.85) |
| **Cholesterol-lowering** | 5 ( 7.5) | 22 (14.1) | 24 (19.8) | 23 (24.7) | 0.002* | 4.85 (1.77-13.31) * |

OR indicates odds ratio; CI, confidence interval; and TIA, transient ischaemic attack. † Cochran-Armitage tests for trend. ‡ 2012-15 with reference to 2000-03 after adjusting for age, sex, and ethnicity and allowing for an interaction between time and ethnicity as appropriate.

**Fig G: Prior risk factors and medication use over time in patients with first-ever ischaemic stroke at <55 years.**





P-values were obtained for the unadjusted rates by the Cochran-Armitage tests for trend. Adjusted rates were computed by multiplying the adjusted coefficients in each time period by the baseline rate in the 2000-2003 cohort. Adjustments were made for age, sex, and ethnicity with an interaction term between time in years and ethnicity as appropriate.

# Trends in risk factors in patients 55+ years

**Table J: Premorbid risk factors and medication use in patients with an incident ischaemic stroke at age 55+ years.**

|  | **2000-2003 (n=725)** | **2004-2007 (n=960)** | **2008-2011 (n=494)** | **2012-2015 (n=450)** | **P-value (trend)†** | **Adjusted model‡** |
| --- | --- | --- | --- | --- | --- | --- |
|  |  |  |  |  |  | **OR (95% CI)** |
| ***Premorbid risk factors*** | | | | | | |
| **Current drinker** | 350 (51.6) | 491 (57.2) | 187 (43.8) | 174 (43.6) | 0.0002* | 0.83 (0.59-1.16) |
| **Smoker** | 190 (28.4) | 240 (26.9) | 123 (27.6) | 90 (22.0) | 0.045* | 0.62 (0.43-0.91) * |
| **Hypertension** | 455 (63.7) | 690 (72.3) | 346 (72.1) | 332 (74.9) | <0.0001* | 1.55 (1.18-2.02) * |
| **Diabetes mellitus** | 145 (20.4) | 231 (24.3) | 125 (25.8) | 119 (27.0) | 0.007* | 1.21 (0.83-1.77) |
| **Hypercholesterolaemia** | 107 (15.3) | 271 (28.4) | 177 (37.1) | 221 (50.3) | <0.0001* | 5.58 (4.22-7.4) * |
| **Atrial fibrillation** | 139 (19.4) | 164 (17.2) | 88 (18.4) | 117 (26.6) | 0.007* | 1.66 (1.19-2.32) * |
| **Myocardial infarction** | 90 (12.6) | 106 (11.1) | 36 ( 7.6) | 48 (11.2) | 0.12 | 0.9 (0.62-1.32) |
| **TIA** | 94 (13.1) | 110 (11.5) | 43 ( 9.0) | 52 (11.8) | 0.21 | 0.97 (0.68-1.4) |
| ***Premorbid medication*** | | | | | | |
| **Antihypertensive** | 353 (50.3) | 548 (57.7) | 175 (37.0) | 174 (39.5) | <0.0001* | 0.58 (0.45-0.74) * |
| **Anti-diabetic** | 145 (20.1) | 234 (24.5) | 100 (20.6) | 100 (22.7) | 0.6 | 0.89 (0.6-1.34) |
| **Antiplatelet** | 249 (44.9) | 406 (48.0) | 208 (44.3) | 149 (33.9) | 0.0003* | 0.71 (0.55-0.92) * |
| **Anticoagulant** | 26 ( 4.8) | 40 ( 4.2) | 24 ( 5.1) | 30 ( 6.8) | 0.11 | 1.63 (0.93-2.84) |
| **Cholesterol-lowering** | 86 (14.5) | 276 (29.1) | 204 (43.1) | 177 (40.1) | <0.0001* | 4.41 (3.27-5.96) * |

OR indicates odds ratio; CI, confidence interval; and TIA, transient ischaemic attack. † Cochran-Armitage tests for trend. ‡ 2012-15 with reference to 2000-03 after adjusting for age, sex, and ethnicity and allowing for an interaction between time and ethnicity as appropriate.

**Fig H: Prior risk factors and medication use over time in patients with first-ever ischaemic stroke at 55+ years.**





P-values were obtained for the unadjusted rates by the Cochran-Armitage tests for trend. Adjusted rates were computed by multiplying the adjusted coefficients in each time period by the baseline rate in the 2000-2003 cohort. Adjustments were made for age, sex, and ethnicity with an interaction term between time in years and ethnicity as appropriate.

# Trends in risk factors in patients with LAA

**Table K: Premorbid risk factors and medication use in patients with an incident LAA stroke.**

|  | **2000-2003 (n=66)** | **2004-2007 (n=132)** | **2008-2011 (n=102)** | **2012-2015 (n=47)** | **P-value (trend)†** | **Adjusted model‡** |
| --- | --- | --- | --- | --- | --- | --- |
|  |  |  |  |  |  | **OR (95%CI)** |
| ***Premorbid risk factors*** | | | | | | |
| **Current drinker** | 30 (47.6) | 72 (59.0) | 55 (59.8) | 19 (45.2) | 0.94 | 0.74 (0.31-1.74) |
| **Smoker** | 20 (31.7) | 49 (38.3) | 37 (39.8) | 19 (42.2) | 0.26 | 1.29 (0.54-3.11) |
| **Hypertension** | 42 (65.6) | 91 (68.9) | 68 (68.0) | 30 (63.8) | 0.85 | 1.18 (0.5-2.76) |
| **Diabetes mellitus** | 15 (24.2) | 28 (21.4) | 25 (24.8) | 13 (27.7) | 0.55 | 1.07 (0.42-2.69) |
| **Hypercholesterolaemia** | 8 (12.5) | 37 (28.2) | 31 (30.7) | 21 (45.7) | 0.0003* | 6.45 (2.46-16.91) * |
| **Atrial fibrillation** | 2 ( 3.1) | 2 ( 1.5) | 6 ( 5.9) | 3 ( 6.5) | 0.12 | 2.86 (0.44-18.67) |
| **Myocardial infarction** | 6 ( 9.4) | 12 ( 9.1) | 5 ( 5.1) | 3 ( 6.5) | 0.31 | 0.73 (0.17-3.17) |
| **TIA** | 10 (15.6) | 24 (18.2) | 13 (12.9) | 1 ( 2.2) | 0.032* | 0.14 (0.02-1.16) |
| ***Premorbid medication use*** | | | | | | |
| **Antihypertensive** | 37 (57.8) | 68 (51.5) | 39 (39.0) | 15 (31.9) | 0.001* | 0.38 (0.17-0.88) * |
| **Anti-diabetic** | 15 (23.1) | 28 (21.2) | 19 (18.8) | 13 (27.7) | 0.81 | 1.09 (0.44-2.72) |
| **Antiplatelet** | 22 (46.8) | 51 (44.7) | 35 (35.0) | 14 (29.8) | 0.031* | 0.71 (0.3-1.72) |
| **Anticoagulant** | 0 ( 0.0) | 1 ( 0.8) | 2 ( 2.0) | 2 ( 4.3) | 0.14 | NA |
| **Cholesterol-lowering** | 5 (10.6) | 47 (35.6) | 38 (38.0) | 15 (31.9) | 0.04* | 4.59 (1.51-13.99) * |

OR indicates odds ratio; CI, confidence interval; and TIA, transient ischaemic attack. † Cochran-Armitage tests for trend. ‡ 2012-15 with reference to 2000-03 after adjusting for age, sex, and ethnicity and allowing for an interaction between time and ethnicity as appropriate.

**Fig I: Prior risk factors and medication use over time in patients with first-ever LAA stroke.**





P-values were obtained for the unadjusted rates by the Cochran-Armitage tests for trend. Adjusted rates were computed by multiplying the adjusted coefficients in each time period by the baseline rate in the 2000-2003 cohort. Adjustments were made for age, sex, and ethnicity with an interaction term between time in years and ethnicity as appropriate.

# Trends in risk factors in patients with CE

**Table L: Premorbid risk factors and medication use in patients with an incident CE stroke.**

|  | **2000-2003 (n=225)** | **2004-2007 (n=275)** | **2008-2011 (n=141)** | **2012-2015 (n=161)** | **P-value (trend)†** | **Adjusted model‡** |
| --- | --- | --- | --- | --- | --- | --- |
|  |  |  |  |  |  | **OR (95%CI)** |
| ***Premorbid risk factors*** | | | | | | |
| **Current drinker** | 110 (52.9) | 147 (61.5) | 52 (44.8) | 61 (45.5) | 0.038* | 0.82 (0.47-1.41) |
| **Smoker** | 41 (20.0) | 54 (21.7) | 28 (22.8) | 24 (17.3) | 0.66 | 0.77 (0.43-1.39) |
| **Hypertension** | 150 (67.3) | 187 (68.0) | 94 (69.1) | 125 (77.6) | 0.036* | 1.7 (1.05-2.75) * |
| **Diabetes mellitus** | 40 (17.9) | 51 (18.6) | 35 (25.2) | 28 (17.9) | 0.58 | 0.99 (0.5-1.93) |
| **Hypercholesterolaemia** | 31 (14.4) | 78 (28.4) | 47 (34.6) | 79 (50.0) | <0.0001* | 6.19 (3.76-10.18) * |
| **Atrial fibrillation** | 99 (45.0) | 109 (39.6) | 61 (45.2) | 81 (50.9) | 0.17 | 1.51 (0.98-2.33) |
| **Myocardial infarction** | 44 (19.8) | 50 (18.2) | 7 ( 5.2) | 28 (18.4) | 0.14 | 0.84 (0.49-1.44) |
| **TIA** | 34 (15.2) | 27 ( 9.8) | 11 ( 8.1) | 25 (15.8) | 0.92 | 1.06 (0.56-2.04) |
| ***Premorbid medication use*** | | | | | | |
| **Antihypertensive** | 114 (52.5) | 158 (58.1) | 40 (30.3) | 57 (36.8) | <0.0001* | 0.49 (0.29-0.82) * |
| **Anti-diabetic** | 40 (17.8) | 52 (19.0) | 29 (20.9) | 23 (14.7) | 0.6 | 0.77 (0.38-1.58) |
| **Antiplatelet** | 86 (48.9) | 123 (51.7) | 67 (50.4) | 58 (37.7) | 0.042* | 0.71 (0.46-1.11) |
| **Anticoagulant** | 17 ( 9.7) | 24 ( 8.8) | 13 ( 9.8) | 18 (11.7) | 0.48 | 1.19 (0.59-2.4) |
| **Cholesterol-lowering** | 26 (13.9) | 77 (28.3) | 57 (43.2) | 66 (42.6) | <0.0001* | 4.89 (2.9-8.22) * |

OR indicates odds ratio; CI, confidence interval; and TIA, transient ischaemic attack. † Cochran-Armitage tests for trend. ‡ 2012-15 with reference to 2000-03 after adjusting for age, sex, and ethnicity and allowing for an interaction between time and ethnicity as appropriate.

**Fig J: Prior risk factors and medication use over time in patients with first-ever CE stroke.**





P-values were obtained for the unadjusted rates by the Cochran-Armitage tests for trend. Adjusted rates were computed by multiplying the adjusted coefficients in each time period by the baseline rate in the 2000-2003 cohort. Adjustments were made for age, sex, and ethnicity with an interaction term between time in years and ethnicity as appropriate.

# Trends in risk factors in patients with SVO

**Table M: Premorbid risk factors and medication use in patients with an incident SVO stroke.**

|  | **2000-2003 (n=226)** | **2004-2007 (n=266)** | **2008-2011 (n=166)** | **2012-2015 (n=127)** | **P-value (trend)†** | **Adjusted model‡** |
| --- | --- | --- | --- | --- | --- | --- |
|  |  |  |  |  |  | **OR (95%CI)** |
| ***Premorbid risk factors*** | | | | | | |
| **Current drinker** | 126 (57.3) | 144 (58.8) | 65 (42.5) | 54 (43.5) | 0.0008* | 0.52 (0.32-0.83) * |
| **Smoker** | 87 (39.5) | 77 (30.3) | 43 (27.2) | 30 (24.2) | 0.002* | 0.43 (0.25-0.74) * |
| **Hypertension** | 137 (60.9) | 193 (72.6) | 114 (69.9) | 95 (74.8) | 0.011* | 1.8 (1.1-2.97) * |
| **Diabetes mellitus** | 44 (19.9) | 61 (23.2) | 43 (26.4) | 38 (30.2) | 0.022* | 2.22 (1.07-4.63) * |
| **Hypercholesterolaemia** | 36 (16.5) | 78 (29.4) | 66 (41.0) | 59 (46.5) | <0.0001* | 4.4 (2.67-7.27) * |
| **Atrial fibrillation** | 4 ( 1.8) | 11 ( 4.2) | 10 ( 6.2) | 21 (16.9) | <0.0001* | 10.71 (3.57-32.12) * |
| **Myocardial infarction** | 10 ( 4.5) | 16 ( 6.0) | 10 ( 6.3) | 10 ( 8.1) | 0.18 | 1.82 (0.73-4.56) |
| **TIA** | 21 ( 9.3) | 24 ( 9.0) | 14 ( 8.7) | 13 (10.2) | 0.86 | 0.95 (0.39-2.32) |
| ***Premorbid medication use*** | | | | | | |
| **Antihypertensive** | 93 (42.1) | 144 (54.3) | 59 (37.6) | 51 (40.5) | 0.24 | 0.81 (0.51-1.29) |
| **Anti-diabetic** | 44 (19.6) | 61 (22.9) | 36 (22.0) | 29 (23.0) | 0.49 | 1.56 (0.72-3.38) |
| **Antiplatelet** | 60 (37.3) | 95 (43.0) | 56 (36.4) | 40 (31.7) | 0.19 | 0.84 (0.5-1.4) |
| **Anticoagulant** | 1 ( 0.6) | 3 ( 1.1) | 3 ( 1.9) | 8 ( 6.3) | 0.0009* | 9.43 (1.15-77.06) * |
| **Cholesterol-lowering** | 27 (14.6) | 67 (25.2) | 60 (38.0) | 43 (34.1) | <0.0001* | 3.69 (2.13-6.4) * |

OR indicates odds ratio; CI, confidence interval; and TIA, transient ischaemic attack. † Cochran-Armitage tests for trend. ‡ 2012-15 with reference to 2000-03 after adjusting for age, sex, and ethnicity and allowing for an interaction between time and ethnicity as appropriate.

**Fig K: Prior risk factors and medication use over time in patients with first-ever SVO stroke.**





P-values were obtained for the unadjusted rates by the Cochran-Armitage tests for trend. Adjusted rates were computed by multiplying the adjusted coefficients in each time period by the baseline rate in the 2000-2003 cohort. Adjustments were made for age, sex, and ethnicity with an interaction term between time in years and ethnicity as appropriate.

# Trends in risk factors in patients with UND

**Table N: Premorbid risk factors and medication use in patients with an incident UND stroke.**

|  | **2000-2003 (n=265)** | **2004-2007 (n=407)** | **2008-2011 (n=204)** | **2012-2015 (n=196)** | **P-value (trend)†** | **Adjusted model‡** |
| --- | --- | --- | --- | --- | --- | --- |
|  |  |  |  |  |  | **OR (95%CI)** |
| ***Premorbid risk factors*** | | | | | | |
| **Current drinker** | 119 (49.2) | 198 (55.0) | 73 (41.7) | 76 (42.9) | 0.033* | 0.66 (0.43-1.04) |
| **Smoker** | 70 (29.7) | 111 (29.8) | 56 (29.9) | 42 (23.5) | 0.2 | 0.66 (0.41-1.06) |
| **Hypertension** | 144 (56.0) | 272 (67.7) | 127 (63.5) | 122 (64.6) | 0.14 | 1.35 (0.9-2.02) |
| **Diabetes mellitus** | 52 (20.3) | 105 (26.1) | 44 (21.9) | 54 (28.0) | 0.17 | 1.16 (0.73-1.83) |
| **Hypercholesterolaemia** | 39 (15.5) | 98 (24.3) | 54 (27.6) | 92 (48.7) | <0.0001* | 5.5 (3.46-8.72) * |
| **Atrial fibrillation** | 39 (14.9) | 48 (11.9) | 16 ( 8.1) | 20 (10.5) | 0.06 | 1.04 (0.58-1.87) |
| **Myocardial infarction** | 32 (12.4) | 30 ( 7.5) | 16 ( 8.1) | 13 ( 7.1) | 0.07 | 0.71 (0.35-1.42) |
| **TIA** | 30 (11.7) | 39 ( 9.7) | 18 ( 9.1) | 16 ( 8.5) | 0.25 | 0.93 (0.48-1.78) |
| ***Premorbid medication use*** | | | | | | |
| **Antihypertensive** | 118 (46.5) | 211 (52.8) | 63 (31.8) | 66 (34.6) | <0.0001* | 0.54 (0.36-0.81) * |
| **Anti-diabetic** | 52 (19.9) | 107 (26.4) | 37 (18.4) | 45 (23.3) | 0.95 | 0.89 (0.56-1.44) |
| **Antiplatelet** | 83 (40.5) | 150 (42.9) | 62 (31.5) | 48 (25.1) | <0.0001* | 0.65 (0.42-1.01) |
| **Anticoagulant** | 11 ( 5.4) | 13 ( 3.2) | 7 ( 3.6) | 6 ( 3.1) | 0.32 | 0.81 (0.29-2.29) |
| **Cholesterol-lowering** | 32 (14.4) | 102 (25.5) | 72 (36.5) | 73 (38.0) | <0.0001* | 4.41 (2.71-7.18) * |

OR indicates odds ratio; CI, confidence interval; and TIA, transient ischaemic attack. † Cochran-Armitage tests for trend. ‡ 2012-15 with reference to 2000-03 after adjusting for age, sex, and ethnicity and allowing for an interaction between time and ethnicity as appropriate.

**Fig L: Prior risk factors and medication use over time in patients with first-ever UND stroke.**





P-values were obtained for the unadjusted rates by the Cochran-Armitage tests for trend. Adjusted rates were computed by multiplying the adjusted coefficients in each time period by the baseline rate in the 2000-2003 cohort. Adjustments were made for age, sex, and ethnicity with an interaction term between time in years and ethnicity as appropriate.
