# Supplementary material for: Long-term trends in incidence and risk factors for ischaemic stroke subtypes: Prospective population study of the South London Stroke Register
Source: PLoS Med. 2018 Oct 5;15(10):e1002669. doi: 10.1371/journal.pmed.1002669 (PMC6173399; doi:10.1371/journal.pmed.1002669)
Supplement: S1 Text — (DOCX) [file pmed.1002669.s003.docx]

**Analysis Plan**

**Long-Term Trends in Incidence and Risk Factors for Ischaemic Stroke Subtypes: Prospective Population Study of The South London Stroke Register**

Hatem A Wafa, MPH; Charles D.A. Wolfe, FFPH; Anthony Rudd, FRCP; Yanzhong Wang, PhD

# Introductin and rationale

The incidence of ischaemic stroke has been declining during the past decades,^1-3^ however, recent analyses have shown increasing trends in particular subtypes of ischaemic stroke.^4-6^ Furthermore, it is not clear whether the changes in ischaemic stroke incidence are consistent among the different subtypes of IS since an overall reduction could possibly mask an increase in one or more subtypes. Up to date investigation of trends in the incidence of IS by aetiological subtypes is therefore pertinent and we aim to present these estimates using data from the population-based South London Stroke Register (SLSR). Furthermore, we aim to analyse the corresponding trends in risk factors and medication use to give insights into possible explanations of any observed trends.

# Study design and dataset

The SLSR is a prospective population-based stroke register set up in January 1995, recording all first-ever strokes in a defined region of Lambeth and Southwark, with a population of 310,026 according to the 2001 United Kingdom Census, with 63% white, 28% black (9% black Caribbean, 15% black African, and 4% black other), and 9% other ethnic groups.^7^ At the time of the 2011 census, the SLSR source population had increased to 357,308 with 56% white, 25% black (7% black Caribbean, 14% black African, 4% black other), and 18% other groups. The SLSR methods are described in detail elsewhere and are discussed briefly here.^8,9^ Stroke was defined according to World Health Organization criteria. Standardized criteria were applied for ensuring completeness of case ascertainment, including multiple overlapping sources of information. Data were collected prospectively by specially trained study nurses and field workers. Stroke diagnosis was verified by a study doctor. Hospital surveillance for admission of stroke included hospitals serving the study area. Community surveillance for stroke included patients under the care of all general practitioners within the study area, and general practitioners were contacted regularly and asked to notify all patients with stroke. Notification sources included accident and emergency records, hospital wards, requests for brain imaging, death certificates, coroner’s records, hospital medical staff, community therapists, bereavement officers, and hospital-based stroke registers. Capture–recapture models estimated completeness of case ascertainment in this population to be ≈80% (between 75% and 88%) as shown in the previous studies from SLSR.^10^

# Statistical analyses

## Comparison of baseline measures

Prior to performing analyses to estimate trends in incidence and risk factors, we propose that descriptive and exploratory analyses will be performed to identify and display differences in baseline characteristics between: (1) the types of ischaemic strokes, and (2) every 4-year interval of index cases between 2000-2015. In particular, statistical comparisons of baseline mean (using ANOVA) and prevalences (using chi-squared tests) between the different subtypes of ischaemic strokes, as classified by the trial of Org 10172 in Acute Stroke Treatment (TOAST), will be performed. Furthermore, the differences across time, classified into four levels each is a 4-year interval of index cases between 2000-2015, will also be investigated and presented. The rationale for performing these initial descriptive analyses is because an understanding of how IS patients differ by subtypes and across time might aid the interpretation of any apparent between-subtype and between-period differences that may arise.

## Analysing trends in incidence

The source population data for the SLSR area, by age, sex, and ethnicity, will be obtained from the office of national statistics (ONS) census estimates of 1991, 2001, and 2011. The demographic composition by age, sex, and ethnic groups will be estimated between any two consecutive censuses assuming linear trends taking into account the new boundaries definition introduced by the government in 2001 and the expansion of the SLSR area to include additional sites during the 2004-2007 period. Crude incidence rates will then be calculated by dividing the number of index cases at each year, or time period, by the corresponding mid-year, or mid-period, estimates of the source population. These rates will then be directly standardised to both the 2011 census population of England and Wales and the 2013 European Standard Population with the 95% confidence intervals estimated assuming Poisson distribution for the number of events. These analyses will be performed to obtain an overall rate for ischaemic stroke and subtypes-specific rates by TOAST criteria with further stratification of these by age (<55 and 55+), sex and ethnic groups (white and black).

Trends will then be assessed by applying the Cochran-Armitage methods and Poisson regression models to the age-adjusted and unadjusted rates. The annual age-adjusted incidence rates will be presented graphically in a scatter plot with regression fitted lines by loess regression to display trends. In addition, the magnitude of change in the standardised rates (Poisson coefficients) along with the 95% confidence interval will be shown for the later 2012-2015 period with reference to the earlier 2000-2003 in a forest plot.

## Analysing trends in risk factors

Selected premorbid variables known to be associated with stroke will be investigated for changes over time. These include: smoking (current vs quitter/never), alcohol intake (≥21 units/week for men, ≥14 units/week for women), hypertension (systolic blood pressure >140 mmHg or diastolic >90 mmHg), diabetes mellitus, hypercholesterolemia (total cholesterol concentration ≥6 mmol/L), myocardial infarction, transient ischaemic attacks, and atrial fibrillation. Moreover, changes in the rates of using preventive medication will also be analysed. These are: antihypertensive drugs, anti-diabetic medications (with oral hypoglycaemics or insulin), cholesterol-lowering agents, antiplatelet, and anticoagulants.

Year of stroke variable will be categorised into four levels, each indicates a 4-year interval from 2000 to 2015 and preliminary trend analyses will be conducted by evaluating whether there are significant differences across time periods in the level of each risk factor using chi-squared tests. Trends in proportions will then be assessed by the Cochran-Armitage tests and the resulting P-values will be presented along with the unadjusted rates.

Since the magnitude of change in risk factors level may be confounded by age, sex, and ethnicity and a correlation between time and race could possibly modify the proportional effect of time, logistic regression models will be fitted with simultaneous adjustment for age, sex, ethnicity (handled as appropriate) and a two-way interaction terms of ethnicity with time (i.e. time-by-ethnicity) will be incorporated. Interaction will be regarded as nominally significant if the two-sided P-value is less than 0.05 after adjustments for multiplicity and backward elimination of non-significant terms will be performed.

The results of these analyses (unadjusted and adjusted rates) will be shown graphically in a line plot.

The frequency of missing data for each variable will be assessed. Depending on the extent of missing data, a range of statistical approaches will be used including multiple imputation and complete case analyses. In the unlikely scenario that these analyses give qualitatively different results the reasons for the differences will be explored and reported in publication. Otherwise, primary focus will be based on estimates from multiply imputed datasets.

# References

1. Bennett DA, Krishnamurthi RV, Barker-Collo S, et al. The global burden of ischemic stroke: findings of the GBD 2010 study. *Glob Heart.* 2014;9(1):107-112.

2. Madsen TE, Khoury J, Alwell K, et al. Sex-specific stroke incidence over time in the Greater Cincinnati/Northern Kentucky Stroke Study. *Neurology.* 2017;89(10):990-996.

3. Rothwell PM, Coull AJ, Giles MF, et al. Change in stroke incidence, mortality, case-fatality, severity, and risk factors in Oxfordshire, UK from 1981 to 2004 (Oxford Vascular Study). *Lancet.* 2004;363(9425):1925-1933.

4. Benatru I, Rouaud O, Durier J, et al. Stable stroke incidence rates but improved case-fatality in Dijon, France, from 1985 to 2004. *Stroke.* 2006;37(7):1674-1679.

5. Kolominsky-Rabas PL, Wiedmann S, Weingartner M, et al. Time trends in incidence of pathological and etiological stroke subtypes during 16 years: the Erlangen Stroke Project. *Neuroepidemiology.* 2015;44(1):24-29.

6. Krishnamurthi RV, Barker-Collo S, Parag V, et al. Stroke Incidence by Major Pathological Type and Ischemic Subtypes in the Auckland Regional Community Stroke Studies: Changes Between 2002 and 2011. *Stroke.* 2018;49(1):3-10.

7. Wang Y, Rudd AG, Wolfe CD. Age and ethnic disparities in incidence of stroke over time: the South London Stroke Register. *Stroke.* 2013;44(12):3298-3304.

8. Stewart JA, Dundas R, Howard RS, Rudd AG, Wolfe CD. Ethnic differences in incidence of stroke: prospective study with stroke register. *BMJ.* 1999;318(7189):967-971.

9. Wolfe CD, Crichton SL, Heuschmann PU, et al. Estimates of outcomes up to ten years after stroke: analysis from the prospective South London Stroke Register. *PLoS Med.* 2011;8(5):e1001033.

10. Heuschmann PU, Grieve AP, Toschke AM, Rudd AG, Wolfe CD. Ethnic group disparities in 10-year trends in stroke incidence and vascular risk factors: the South London Stroke Register (SLSR). *Stroke.* 2008;39(8):2204-2210.
